# Supplementary material for: Circum-Arctic release of terrestrial carbon varies between regions and sources
Source: Nat Commun. 2022 Oct 4;13:5858. doi: 10.1038/s41467-022-33541-0 (PMC9532443; doi:10.1038/s41467-022-33541-0)
Supplement: Supplementary file 1 — Supplementary Information [file 41467_2022_33541_MOESM1_ESM.pdf]

Supplementary Information

Circum-Arctic release of terrestrial carbon varies between regions and sources

Jannik Martens, Birgit Wild, Igor Semiletov, Oleg V. Dudarev, and Örjan Gustafsson

Supplementary Discussion 1: Extended discussion of terrestrial organic carbon accumulation in the circum-Arctic shelf seas

Based on the massive underlying CASCADE dataset, we estimate that the total terrOC accumulation in all circum-Arctic shelf seas combined is 44.9±18 Tg yr^{-1} (Table S1). This is one order of magnitude higher than a previous receptor-based estimate (5.5 Tg yr^{-1}), deduced from accumulation rates averaged over the Holocene using ^{14}C dating of sediment cores[1]. Our estimate is much more in line with an earlier estimate of accumulation of only the ICD-OC fraction (about half of the total terrOC) in the Laptev and East Siberian seas of 22 Tg yr^{-1} [2]. Surface soil and peat OC are mostly transported by rivers and combine to a receptor-based accumulation flux that is 83% (31.5±18Tg yr^{-1}) of the estimated circum-Arctic fluvial OC discharge to the Arctic Ocean (~38.1 Tg yr^{-1}; Table S1)[3,4]. The offset of ~6.6 Tg yr^{-1} roughly agrees with the estimated CO_2 evasion from terrOC degradation in the Siberian Seas of 4-10 Tg yr^{-1} [5–7].

The receptor-based estimate of the ICD-OC release flux is 13.4±4.6 Tg yr^{-1} and agrees well with a previous study solely based on coastal erosion rates and OC contents of the coast line (15.4 Tg yr^{-1})[8]. For the Laptev and East Siberian seas specifically, this study estimates an ICD-OC release flux (10.5±3.4 Tg yr^{-1}), half that of the earlier estimate by Vonk and co-workers (22 Tg yr^{-1}; ref[2]). These figures are higher than the last circum-Arctic estimate of coastal terrOC input, which is 6.7 Tg yr^{-1} [1].

Supplementary Methods 1: End member definition for the Circum-Arctic shelf seas

To distinguish between terrOC and marine OC this study applied a large database of ^{13}C and ^{14}C measurements of end member materials within the circum-Arctic watershed from the published literature. For all regions, the source apportionment assumes mixing from two terrestrial end members and marine phytoplankton as major OC sources. Overall, terrOC end members represent i) OC in surface soils with 0-100 cm depth, with an age of up to a few thousand years (incl. permafrost soils; except for the Beaufort Sea where surface soil was combined with peat), and ii) a strongly pre-aged terrOC source from deeper layers (ICD, petrogenic C, or deep peat OC; depending on occurrence in the catchment).

Surface soil

The circum-Arctic end member for surface soil builds on a large in-house data collection employed by previous studies[9,10], which was further amended by a large soil radiocarbon database[11], and only contains data of samples from inside the circum-Arctic watershed. The surface soil end member contains data from the permafrost active layer in permafrost regions to a maximum depth of 100 cm, and data for non-permafrost soils to a depth of 100 cm. The $\delta^{13}C$ value of the surface soil end member is -26.6 ± 1.7‰ (n=219) and the $\Delta^{14}C$ is -201.1 ± 229.3‰ (n=304).

Marine organic carbon

This study applied two different marine OC end members. The open-marine OC end member is based on marine phytoplankton/algae sampled from open ocean settings under influence of water mass inflow from the Atlantic or the Pacific Ocean (-50 ± 12‰ for $\Delta^{14}C$; n=5; -21 ± 2.6‰ for $\delta^{13}C$; n=31) and was applied in the Canadian Arctic Archipelago, Beaufort Sea, Chukchi Sea, ESAS east of 160°E, and the Barents Sea. The open marine end member excludes any measurements of phytoplankton from the Laptev and East Siberian seas west of the frontal zone at 160°E[12] and the Kara Sea, since these areas are strongly affected by terrOC and which influences the $\delta^{13}C$ ratios of marine phytoplankton[13,14]. A wider marine end member was consequently applied for the Laptev and Kara Seas, as well as the East Siberian Sea west of 160°E. The wider marine endmember contains all data of marine phytoplankton available

for the Arctic Ocean (-16 ± 53‰ for $\Delta^{14}C$; n=12; -23.2 ± 3.5‰ for $\delta^{13}C$; n=52) and thereby accounts for the possible influence of terrOC on the isotopic composition of marine phytoplankton in this region.

ICD

The end member for ICD in Eastern Siberia, Alaska and northwestern Canada applied a large in-house database of ^{14}C measurements from coastal exposures in Siberia, Alaska and northwestern Canada that was published and updated throughout previous studies[2,9,10]. The $\Delta^{14}C$ of the ICD end member is -960.3 ± 60.7‰ (n=487). For the $\delta^{13}C$, this study applied an average value reported in a literature review (-26.3 ± 0.7‰; n=374)[15].

Peat

This study also accounted for peat as a potential terrOC source to Arctic Ocean sediments. For the Kara Sea catchment, which hosts the world's largest peatland, this study distinguished an end member for peat deposits deeper than 100 cm depth using a large collection of ^{14}C-dated peat cores[16] from the Circum-Arctic (-503.2 ± 158.9‰; n=263). For the Beaufort Sea, this study accounted for the large peatlands in the drainage basin by using a combined end member for surface soil and peat OC, which is based on published ^{13}C and ^{14}C data from peat core samples (incl. 0-100 cm depth) within the watershed of the Beaufort Sea (-377.9 ± 201.3‰; n=191). Data for $\delta^{13}C$ in peat is rare but is expected to resemble the isotopic characteristics of terrOC that was produced by C3 plant photosynthesis (-27‰). This study thus used the same $\delta^{13}C$ for peat as for surface soil OC (-26.6 ± 1.7‰; n=219).

Petrogenic C

A petrogenic carbon end member was applied for the Canadian Arctic Archipelago, the Canadian Beaufort Sea and the Barents Sea to account for ancient C reservoirs in the form of petrogenic sources in these catchments[17]. The petrogenic carbon end member is based on previous studies of sediment OC sources in this region and builds on $\delta^{13}C$ measurements of kerogen in sedimentary rocks around -26‰±2[18,19] while the $\Delta^{14}C$ was assumed to be ^{14}C-dead/undetectable at -998‰.

Supplementary Table 1: Calculated accumulation (mean ± s.d.) of OC fractions per shelf sea in Tg yr^{-1}. The terrOC sources vary between the shelf seas based on their presence in the respective drainage basin. The sources are surface soil (SurfSoil), Ice Complex deposits (ICD), peat and total terrestrial (terr) OC.

	CAA	Beaufort Sea	Chukchi Sea	East Sib Sea	Laptev Sea	Kara Sea	Barents Sea	Total
SurfSoil-OC	2.9 ±2.0	0 - 1.1 ±0.7*	3.1 ±2.0	6.0 ±3.7	3.3 ±2.1	6.2 ±4.1	4.5 ±3.0	27.2 ±17.7
ICD-OC		0.7 ±0.2	2.2 ±1.0	6.7 ±2.2	3.7 ±1.2			13.4 ±4.6
Peat OC		0 - 1.1 ±0.7*				4.3 ±2.6		4.3 ±2.6
**TerrOC**	**2.9 ±2.0**	**1.8 ±0.7**	**5.3 ±2.2**	**12.7 ±4.3**	**7.1 ±2.4**	**10.6 ±4.9**	**4.5 ±3.0**	**44.9 ±18.4**

*Combined for SurfSoil-OC and Peat (further detail provided in Text S1)

Supplementary Table 2: Terrestrial OC stocks in the Circum-Arctic drainage basin (in Pg).

	CAA	Beaufort Sea	Chukchi Sea	East Siberian Sea	Laptev Sea	Kara Sea	Barents Sea	Total
Surface soil[1]	28	55	25	42	93	157	40	439
ICD[2]		6	50	83	63	11		212
Peat[3]	1	12	1	2	4	44	16	80
**Total terrestrial**	**29**	**73**	**75**	**127**	**159**	**212**	**56**	**731**

[1]based on ref[20,21]
[2]based on ref[22]
[3]based on ref[23]

Supplementary Table 3: Results (mean ± s.d.) of the Integrated Carbon Release Index (I-CRI) for the Circum-Arctic shelf seas. The I-CRI distinguishes between surface soil (SurfSoil), Ice Complex deposits (ICD), peat and the total terrestrial (terr) OC.

	CAA	Beaufort Sea	Chukchi Sea	East Sib Sea	Laptev Sea	Kara Sea	Barents Sea
$I\text{-}CRI_{SurfSoil}$	1.0 ±0.7	0.2 ±0.1*	1.2 ±0.8	1.4 ±0.9	0.4 ±0.2	0.4 ±0.3	1.1 ±0.7
$I\text{-}CRI_{ICD}$		1.1 ±0.3	0.4 ±0.2	0.8 ±0.3	0.6 ±0.2		
$I\text{-}CRI_{Peat}$		0.2 ±0.1*				1.0 ±0.2	
**$I\text{-}CRI_{terr}$**	**1.0 ±0.7**	**0.2 ±0.1**	**0.7 ±0.3**	**1.0 ±0.4**	**0.4 ±0.2**	**0.5 ±0.2**	**0.8 ±0.5**

*Combined for SurfSoil-OC and Peat

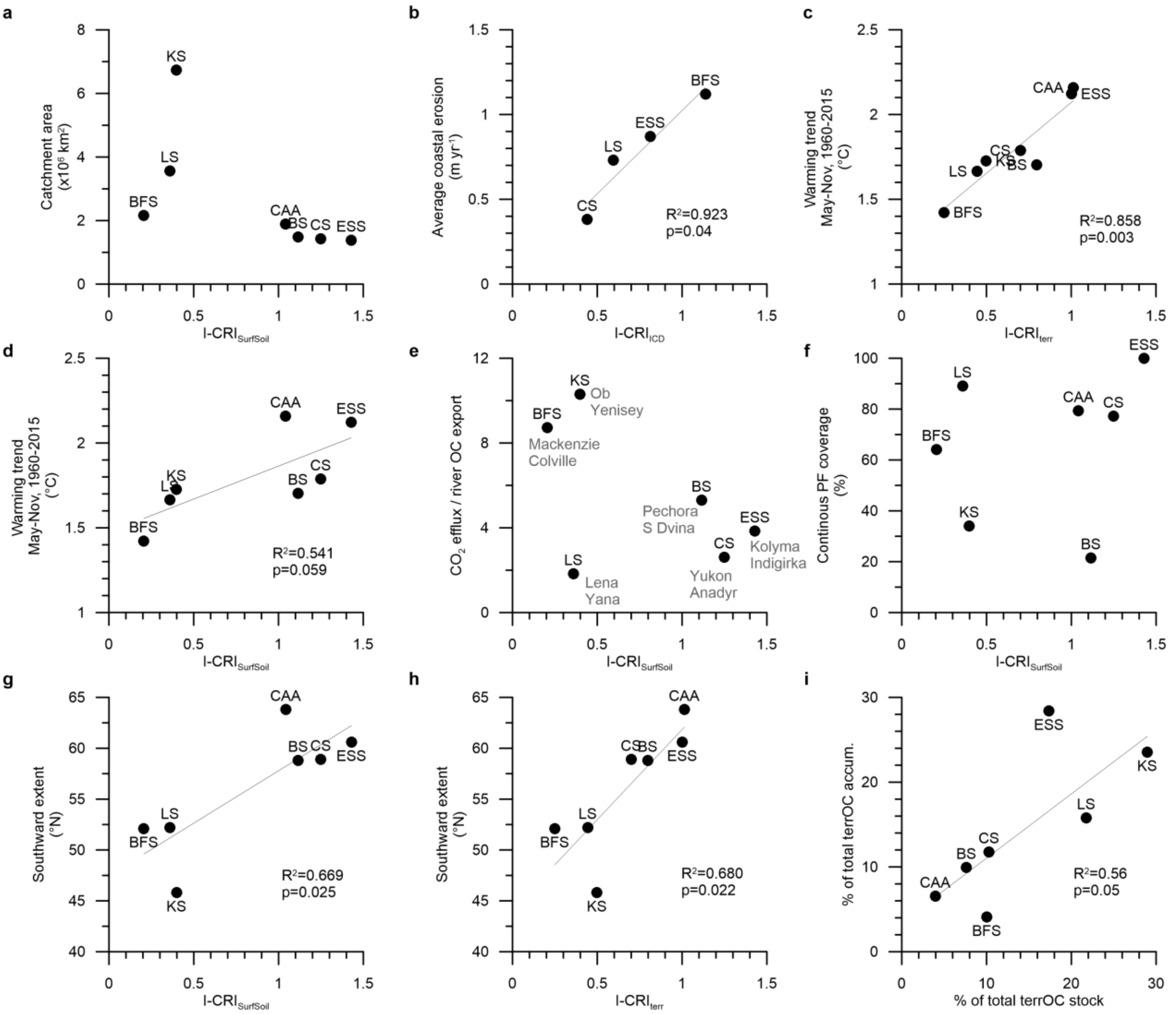

Supplementary Fig. 1: Comparative analysis between the I-CRI of the different terrestrial organic carbon (terrOC) compartments and environmental factors for each drainage basin. Shown are a) the relationship between the $I\text{-}CRI_{SurfSoil}$ and the catchment area, and b) the $I\text{-}CRI_{ICD}$ and average rates of coastal erosion[24]. Further shown are the I-CRI for c) terrOC and d) for surface soil (SurfSoil) with warming trends of the warm period (May-Nov) for 1960-2015[25,26]. Panel e) shows the ratio of estimated inland CO_2 efflux[27] to the total OC discharge for the major river systems (denoted as grey text) of each drainage basin[4,28,29]. Panel f) shows the $I\text{-}CRI_{SurfSoil}$ and the distribution of continous permafrost[30]. Panels g) and h) show the correlation between the $I\text{-}CRI_{SurfSoil}$ and $I\text{-}CRI_{terr}$ with the southward extent of the drainage basins. Panel i) shows the correlation between the % of the total terrOC stock and the % of total terrOC accumulation for each circum-Arctic shelf sea. Solid black lines indicate significant correlations, for which the coefficients of determination (R^2) and p-values are shown. The abbreviations of the shelf seas are CAA – Canadian Arctic Archipelago, BFS – Beaufort Sea, CS – Chukchi Sea, ESS – East Siberian Sea, LS – Laptev Sea, KS – Kara Sea, BS – Barents Sea, CAO – Central Arctic Ocean.

Supplementary Table 4: End members for the dual-isotope mixing model to calculate OC source fractions. The end member definition is detailed in Text S1.

**End members**	**$\delta^{13}C$, ‰**		**$\Delta^{14}C$, ‰**	
	Mean ± s.d.		Mean ± s.d.	
Surface soil OC incl. permafrost active layer; 0-100 cm	-26.6	1.7	-201.1	229.3
Ice Complex Deposits	-26.3	0.7	-960.3	60.7
Petrogenic carbon	-26.0	2.0	-998.0	2
Marine OC	-21.0	2.6	-50	12
Marine OC (Laptev and East Sib Sea west of 160E, Kara Sea)	-23.2	3.5	-3	55
Peat deposits > 1 m depth (Kara Sea)	-26.6	1.7	-503.2	158.9
Surface soil incl. peat deposits (Beaufort Sea)	-26.6	1.7	-377.9	201.3

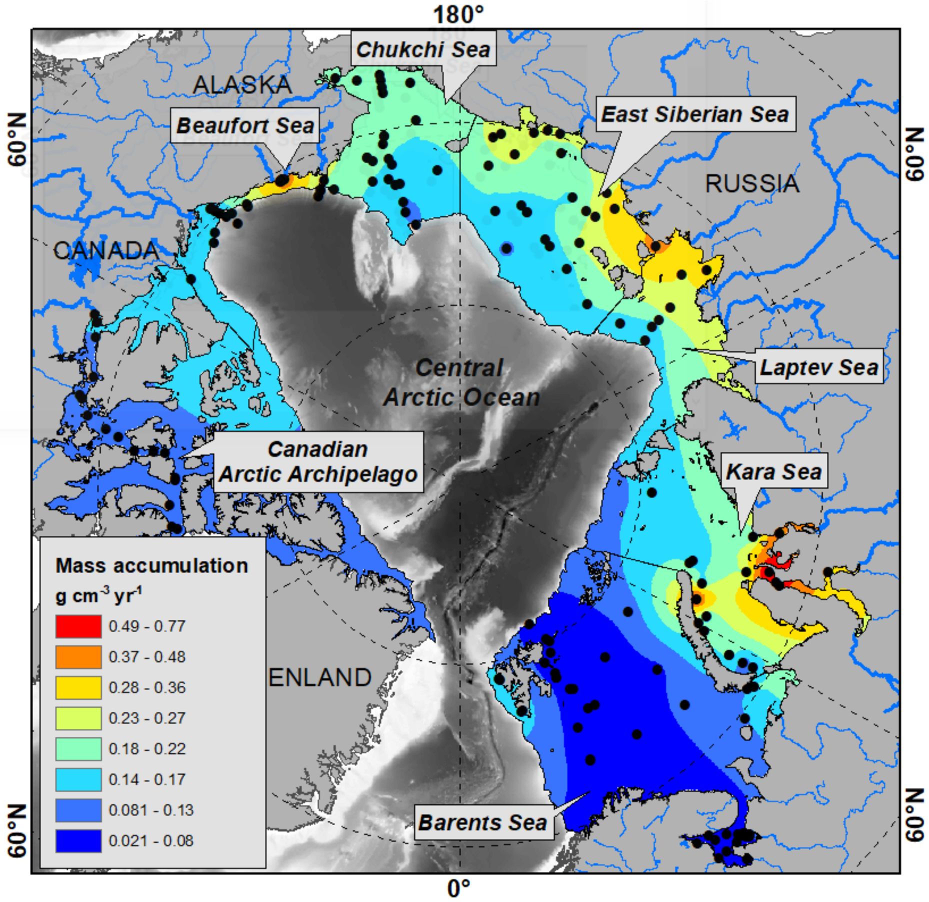

Supplementary Fig. 2: Interpolated mass accumulation rates of surface sediments in the circum-Arctic shelf seas including the locations of ^{210}Pb-dated sediment cores as black dots. The Arctic Ocean base map is based on IBCAOv4[31,32].

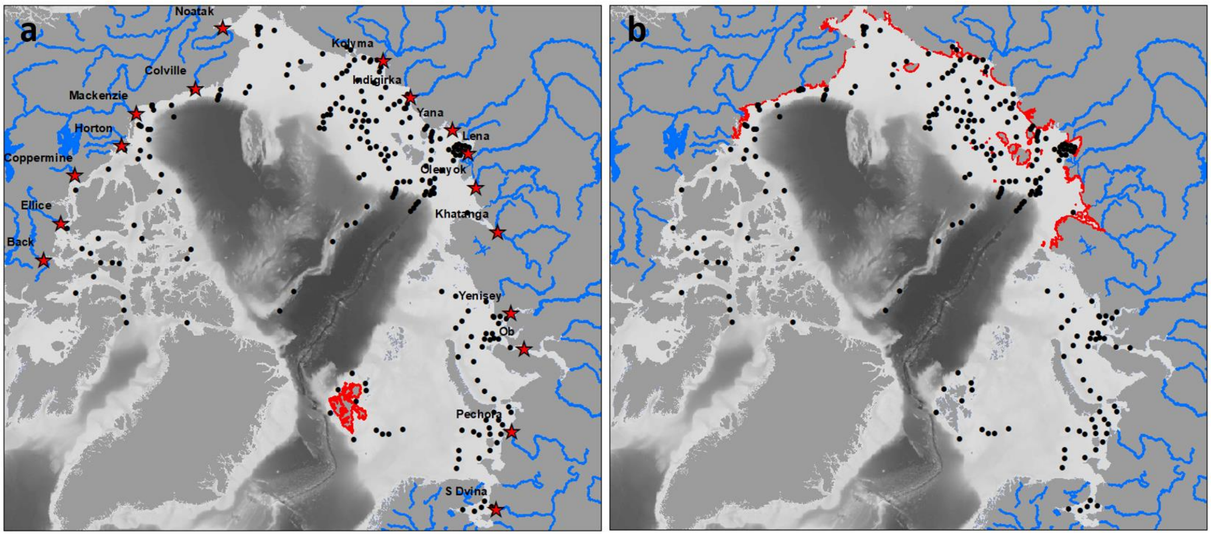

Supplementary Fig. 3: Estimation of cross-shelf transport distances between the sampling points (black dots) and a) river outlets in the circum-Arctic for surface soil and peat OC end members. For Svalbard, the coastline was used instead. Panel b) shows the sites of coastal erosion in areas with ICD present, which was used to estimate the transport distance for the ICD end member. The Arctic Ocean base map is based on IBCAOv4[31,32].

Supplementary Table 5: Results of the dual-isotope source apportionment. Shown are the input dual-isotope data from CASCADE and the source fractions of surface soil (SurfSoil), Ice Complex deposits (ICD), peat, marine organic carbon (MOC) and petrogenic carbon.

Station ID	Lat	Long	Shelf Sea*	$\delta^{13}C$ ‰	$\Delta^{14}C$ ‰	SurfSoil	s.d.	ICD	s.d.	Peat	s.d.	MOC	s.d.	Petro	s.d.
QM1	68.667	-103.004	CAA	-21.7	-127.9	0.20	0.14	0.00	0.00	0.00	0.00	0.73	0.14	0.06	0.03
VS1	70.249	-98.929	CAA	-21	-182.9	0.18	0.12	0.00	0.00	0.00	0.00	0.73	0.10	0.09	0.04
FS1	70.999	-97.998	CAA	-21.7	-826.9	0.10	0.08	0.00	0.00	0.00	0.00	0.12	0.05	0.78	0.05
BE2	71.973	-95.998	CAA	-21.1	-754.2	0.09	0.09	0.00	0.00	0.00	0.00	0.21	0.05	0.70	0.06
PS1	73.597	-96.295	CAA	-20.5	-301.8	0.17	0.13	0.00	0.00	0.00	0.00	0.65	0.07	0.18	0.07
PS2	73.000	-96.218	CAA	-21.5	-887.1	0.08	0.07	0.00	0.00	0.00	0.00	0.08	0.03	0.84	0.05
CAA2	73.935	-86.108	CAA	-22	-726.3	0.14	0.15	0.00	0.00	0.00	0.00	0.23	0.06	0.63	0.10
CAA1	73.927	-81.814	CAA	-21.6	-206.3	0.17	0.11	0.00	0.00	0.00	0.00	0.75	0.07	0.08	0.05
BB11	73.933	-77.930	CAA	-22	-181.4	0.14	0.09	0.00	0.00	0.00	0.00	0.79	0.06	0.07	0.04
89200-B7	79.295	-101.904	CAA	-23.9	-842.4	0.22	0.17	0.00	0.00	0.00	0.00	0.10	0.05	0.68	0.14
89200-E4	79.443	-106.603	CAA	-24.9	-793.2	0.31	0.21	0.00	0.00	0.00	0.00	0.12	0.06	0.57	0.16
2006804-002A	79.000	-72.285	CAA	-23.7	-521.5	0.33	0.20	0.00	0.00	0.00	0.00	0.40	0.08	0.27	0.14
2006804-013A	71.482	-102.235	CAA	-22.7	-723.5	0.15	0.14	0.00	0.00	0.00	0.00	0.21	0.07	0.65	0.09
2009804-068	74.101	-108.834	CAA	-25.6	-507.3	0.48	0.23	0.00	0.00	0.00	0.00	0.26	0.13	0.26	0.13
9722-006E	75.065	-105.347	CAA	-22.6	-377.3	0.27	0.18	0.00	0.00	0.00	0.00	0.52	0.10	0.21	0.09
2006804-016A	70.100	-120.054	CAA	-26.2	-501.6	0.48	0.21	0.00	0.00	0.00	0.00	0.20	0.13	0.31	0.13
2006804-023A	70.340	-126.360	CAA	-25.2	-669.6	0.26	0.17	0.00	0.00	0.00	0.00	0.17	0.09	0.57	0.11
2016-805-021	76.156	-125.875	CAA	-22.2	-588.3	0.18	0.17	0.00	0.00	0.00	0.00	0.35	0.08	0.47	0.11
2016-805-027	74.886	-122.170	CAA	-21.8	-481.6	0.19	0.16	0.00	0.00	0.00	0.00	0.45	0.08	0.36	0.10
2016-805-033	68.385	-112.088	CAA	-22.2	-127	0.23	0.16	0.00	0.00	0.00	0.00	0.71	0.16	0.06	0.03
2017804-0042	71.475	-91.993	CAA	-21.3	-425.8	0.17	0.15	0.00	0.00	0.00	0.00	0.51	0.08	0.31	0.09
2017804-0046	69.955	-87.667	CAA	-20.1	-182.5	0.15	0.10	0.00	0.00	0.00	0.00	0.76	0.08	0.09	0.04
G7	69.892	-133.412	BFS	-25.5	-691.3	0.33	0.20	0.00	0.00	0.00	0.00	0.16	0.09	0.51	0.15
GRM1	70.017	-133.433	BFS	-25.7	-720.8	0.31	0.20	0.00	0.00	0.00	0.00	0.14	0.08	0.55	0.15
G12	70.737	-134.165	BFS	-24.4	-590.9	0.33	0.21	0.00	0.00	0.00	0.00	0.26	0.11	0.40	0.14
G10	70.947	-134.595	BFS	-25.5	-876.8	0.16	0.14	0.00	0.00	0.00	0.00	0.06	0.04	0.77	0.12
Station 5/G7	70.170	-133.433	BFS	-26	-737.8	0.31	0.19	0.00	0.00	0.00	0.00	0.13	0.08	0.56	0.15
Station 9/G12	70.737	-134.165	BFS	-24.9	-618.6	0.36	0.21	0.00	0.00	0.00	0.00	0.22	0.11	0.42	0.14
Station 35	71.408	-132.333	BFS	-24.9	-726.8	0.29	0.20	0.00	0.00	0.00	0.00	0.16	0.08	0.55	0.15
Station SS2	71.462	-128.563	BFS	-24.8	-681.6	0.31	0.20	0.00	0.00	0.00	0.00	0.19	0.09	0.51	0.14
Station SS4	69.935	-138.573	BFS	-25.4	-722.1	0.30	0.20	0.00	0.00	0.00	0.00	0.15	0.08	0.55	0.15
2009804-064	71.779	-126.475	BFS	-23.4	-496.8	0.30	0.21	0.00	0.00	0.00	0.00	0.37	0.11	0.33	0.12
2016-805-028	74.179	-126.823	BFS	-22.1	-476.7	0.23	0.18	0.00	0.00	0.00	0.00	0.45	0.08	0.32	0.11
2016-805-030	72.392	-128.952	BFS	-23	-440.6	0.29	0.20	0.00	0.00	0.00	0.00	0.44	0.11	0.27	0.11
PG2303-7	69.513	-138.985	BFS	-26.2	-826.4	0.22	0.17	0.00	0.00	0.00	0.00	0.09	0.05	0.69	0.14
PG2302-1	69.546	-138.921	BFS	-26.6	-629	0.45	0.22	0.00	0.00	0.00	0.00	0.16	0.10	0.40	0.15
PG2305-1	69.567	-138.936	BFS	-25.9	-802.6	0.25	0.18	0.00	0.00	0.00	0.00	0.10	0.06	0.65	0.14
PG2307-1	69.492	-138.953	BFS	-26.4	-681.6	0.39	0.21	0.00	0.00	0.00	0.00	0.14	0.09	0.47	0.15
PG2308-1	69.540	-138.917	BFS	-26.2	-692.5	0.37	0.21	0.00	0.00	0.00	0.00	0.14	0.09	0.49	0.15
PG2312-1	69.556	-138.924	BFS	-25.9	-806.6	0.24	0.17	0.00	0.00	0.00	0.00	0.10	0.06	0.66	0.14
PG2313-1	69.574	-138.939	BFS	-25.5	-773.8	0.26	0.18	0.00	0.00	0.00	0.00	0.12	0.07	0.62	0.14

PG2315-1	69.504	-138.916	BFS	-26.2	-809.5	0.25	0.18	0.00	0.00	0.00	0.00	0.09	0.06	0.66	0.15
PG2316-1	69.496	-138.936	BFS	-26.1	-760.9	0.29	0.19	0.00	0.00	0.00	0.00	0.12	0.07	0.59	0.15
PG2318-1	69.491	-138.959	BFS	-26.2	-733.1	0.33	0.20	0.00	0.00	0.00	0.00	0.13	0.08	0.54	0.15
YC16-LC4	69.484	-138.992	BFS	-25.9	-673.6	0.37	0.21	0.00	0.00	0.00	0.00	0.16	0.09	0.47	0.15
YC16-SC20	69.483	-138.994	BFS	-26.2	-557.5	0.48	0.22	0.00	0.00	0.00	0.00	0.20	0.12	0.32	0.14
BC3	71.578	-156.018	BFS	-22.4	-146.6	0.23	0.16	0.07	0.04	0.00	0.00	0.70	0.15	0.00	0.00
BC4	71.930	-154.887	BFS	-24.6	-571	0.28	0.20	0.46	0.13	0.00	0.00	0.26	0.11	0.00	0.00
BC5	72.000	-154.708	BFS	-24	-546.6	0.25	0.19	0.44	0.12	0.00	0.00	0.31	0.10	0.00	0.00
BC6	72.233	-154.037	BFS	-25.1	-658.6	0.24	0.17	0.57	0.12	0.00	0.00	0.19	0.09	0.00	0.00
CG1	70.562	-142.858	BFS	-23.6	-520.6	0.24	0.20	0.41	0.12	0.00	0.00	0.35	0.10	0.00	0.00
Station 144	71.433	-141.400	BFS	-23.8	-725.4	0.15	0.19	0.63	0.14	0.00	0.00	0.22	0.07	0.00	0.00
ND	70.677	-150.498	BFS	-26.5	-841.2	0.13	0.11	0.81	0.10	0.00	0.00	0.06	0.04	0.00	0.00
L1	70.525	-150.202	BFS	-26.5	-622.7	0.35	0.17	0.53	0.14	0.00	0.00	0.12	0.09	0.00	0.00
L2	70.496	-150.061	BFS	-26.6	-672.4	0.31	0.16	0.59	0.14	0.00	0.00	0.10	0.08	0.00	0.00
L3	70.531	-149.948	BFS	-26.5	-583	0.39	0.18	0.48	0.14	0.00	0.00	0.13	0.10	0.00	0.00
L4	70.537	-149.882	BFS	-26.7	-652.5	0.34	0.17	0.56	0.14	0.00	0.00	0.10	0.08	0.00	0.00
L5	70.541	-149.647	BFS	-26.2	-692.2	0.26	0.15	0.63	0.12	0.00	0.00	0.11	0.08	0.00	0.00
L6	70.524	-149.379	BFS	-26.5	-622.7	0.36	0.17	0.52	0.14	0.00	0.00	0.12	0.09	0.00	0.00
4-PC1	72.839	-175.727	CS	-22.4	-259	0.22	0.14	0.12	0.07	0.00	0.00	0.66	0.09	0.00	0.00
UTN3	67.334	-169.000	CS	-20.6	-214.7	0.17	0.12	0.12	0.05	0.00	0.00	0.71	0.09	0.00	0.00
UTN5	67.670	-168.958	CS	-21.2	-190.8	0.19	0.13	0.10	0.05	0.00	0.00	0.72	0.10	0.00	0.00
UTN7	68.000	-168.933	CS	-21.1	-171.1	0.18	0.12	0.08	0.04	0.00	0.00	0.74	0.10	0.00	0.00
CL-8	67.869	-172.548	CS	-22.4	-250.6	0.25	0.16	0.13	0.06	0.00	0.00	0.62	0.12	0.00	0.00
CL-3	69.003	-168.894	CS	-22.7	-326.9	0.27	0.18	0.20	0.08	0.00	0.00	0.53	0.12	0.00	0.00
HC-2	70.900	-175.013	CS	-22.1	-246.4	0.22	0.14	0.12	0.06	0.00	0.00	0.66	0.10	0.00	0.00
H-4	72.537	-162.248	CS	-22.8	-389.6	0.27	0.19	0.25	0.10	0.00	0.00	0.48	0.11	0.00	0.00
H-6	72.164	-163.603	CS	-23.3	-360.9	0.31	0.20	0.21	0.09	0.00	0.00	0.48	0.13	0.00	0.00
HC-26	71.788	-174.395	CS	-22.1	-287.3	0.23	0.15	0.15	0.07	0.00	0.00	0.62	0.09	0.00	0.00
CEN1A	70.709	-178.299	CS	-23.4	-388.8	0.31	0.19	0.21	0.10	0.00	0.00	0.47	0.11	0.00	0.00
CL6R	67.431	-169.603	CS	-22.1	-255.5	0.23	0.16	0.15	0.06	0.00	0.00	0.62	0.12	0.00	0.00
YS-31	71.592	161.690	ESS	-26.5	-623.6	0.40	0.18	0.48	0.14	0.00	0.00	0.12	0.09	0.00	0.00
YS-32	70.567	161.217	ESS	-26.4	-584.3	0.40	0.18	0.46	0.14	0.00	0.00	0.14	0.10	0.00	0.00
YS-33	70.168	161.217	ESS	-26.2	-514.3	0.43	0.19	0.39	0.13	0.00	0.00	0.17	0.12	0.00	0.00
YS-34B	69.708	162.689	ESS	-27.3	-553.9	0.52	0.18	0.38	0.15	0.00	0.00	0.10	0.09	0.00	0.00
YS-35	69.817	164.057	ESS	-26.8	-542	0.48	0.18	0.39	0.14	0.00	0.00	0.13	0.10	0.00	0.00
YS-36	69.817	165.999	ESS	-26	-546.8	0.41	0.19	0.41	0.14	0.00	0.00	0.18	0.11	0.00	0.00
YS-37	70.135	168.007	ESS	-25.7	-489.2	0.44	0.21	0.33	0.13	0.00	0.00	0.23	0.13	0.00	0.00
YS-38	70.698	169.132	ESS	-25.3	-461.6	0.43	0.21	0.30	0.12	0.00	0.00	0.27	0.14	0.00	0.00
YS-39	71.219	169.373	ESS	-24.3	-427	0.36	0.21	0.28	0.11	0.00	0.00	0.36	0.14	0.00	0.00
YS-40	71.483	170.553	ESS	-23.9	-425	0.33	0.21	0.28	0.11	0.00	0.00	0.39	0.13	0.00	0.00
YS-41	71.968	171.792	ESS	-23.9	-373.3	0.35	0.21	0.21	0.10	0.00	0.00	0.43	0.14	0.00	0.00
YS-86	75.301	174.396	ESS	-21.2	-391.5	0.20	0.16	0.26	0.10	0.00	0.00	0.55	0.07	0.00	0.00
YS-88	75.099	172.187	ESS	-21.5	-446.8	0.19	0.17	0.32	0.11	0.00	0.00	0.49	0.07	0.00	0.00
YS-90	74.668	172.388	ESS	-22.4	-331.6	0.26	0.17	0.18	0.09	0.00	0.00	0.56	0.10	0.00	0.00
YS-93	74.419	165.999	ESS	-23.8	-398.1	0.34	0.21	0.23	0.10	0.00	0.00	0.43	0.13	0.00	0.00

YS-95	74.417	161.335	ESS	-24.8	-511.2	0.37	0.22	0.34	0.13	0.00	0.00	0.29	0.12	0.00	0.00
YS-98	75.551	160.751	ESS	-23.9	-566.2	0.27	0.21	0.42	0.14	0.00	0.00	0.31	0.10	0.00	0.00
YS-99	75.171	163.586	ESS	-23.4	-498.3	0.28	0.21	0.35	0.13	0.00	0.00	0.38	0.11	0.00	0.00
YS-100	75.716	164.079	ESS	-22.4	-450.6	0.23	0.19	0.31	0.11	0.00	0.00	0.45	0.09	0.00	0.00
YS-102	76.559	160.073	ESS	-22.3	-476.1	0.22	0.19	0.35	0.12	0.00	0.00	0.44	0.09	0.00	0.00
SW-52	75.001	161.032	ESS	-23.9	-545.9	0.28	0.21	0.40	0.13	0.00	0.00	0.32	0.10	0.00	0.00
SW-57	74.425	163.692	ESS	-24.2	-296.4	0.37	0.20	0.14	0.08	0.00	0.00	0.49	0.16	0.00	0.00
SW-58	74.439	166.047	ESS	-23.8	-307.4	0.34	0.20	0.15	0.08	0.00	0.00	0.51	0.15	0.00	0.00
SW-59	74.426	168.493	ESS	-23.5	-465.6	0.30	0.21	0.30	0.12	0.00	0.00	0.39	0.11	0.00	0.00
SW-60	73.520	169.460	ESS	-24	-308.3	0.36	0.20	0.15	0.08	0.00	0.00	0.49	0.15	0.00	0.00
SW-61	74.106	170.901	ESS	-24.2	-239.3	0.32	0.20	0.10	0.06	0.00	0.00	0.58	0.16	0.00	0.00
SW-63	74.682	172.369	ESS	-22.7	-289.6	0.26	0.16	0.14	0.07	0.00	0.00	0.59	0.11	0.00	0.00
SW-65	75.163	173.191	ESS	-20.9	-278	0.19	0.13	0.15	0.07	0.00	0.00	0.66	0.07	0.00	0.00
SW-66	75.838	174.409	ESS	-21	-448.3	0.17	0.16	0.32	0.11	0.00	0.00	0.50	0.07	0.00	0.00
lv78-60	73.367	162.769	ESS	-25.3	-676.6	0.27	0.18	0.56	0.13	0.00	0.00	0.17	0.08	0.00	0.00
lv78-61	72.609	164.345	ESS	-25.4	-730.5	0.22	0.16	0.64	0.12	0.00	0.00	0.14	0.07	0.00	0.00
lv78-62	71.800	166.132	ESS	-24.9	-629.7	0.26	0.19	0.52	0.13	0.00	0.00	0.22	0.09	0.00	0.00
lv78-78	69.175	170.585	ESS	-26.6	-408.3	0.58	0.21	0.21	0.11	0.00	0.00	0.21	0.15	0.00	0.00
lv78-81	69.378	169.417	ESS	-24.4	-400.5	0.38	0.21	0.25	0.11	0.00	0.00	0.37	0.14	0.00	0.00
lv57-8	72.640	175.408	ESS	-23.4	-256.8	0.29	0.18	0.12	0.07	0.00	0.00	0.59	0.13	0.00	0.00
lv57-94	69.995	176.215	ESS	-23.1	-383.1	0.29	0.19	0.23	0.10	0.00	0.00	0.48	0.12	0.00	0.00
IK-71	70.000	164.167	ESS	-26.7	-502.9	0.50	0.19	0.35	0.14	0.00	0.00	0.15	0.12	0.00	0.00
18-MC5	76.409	173.879	ESS	-21.4	-474	0.19	0.17	0.34	0.12	0.00	0.00	0.47	0.07	0.00	0.00
21-MC6	77.579	163.308	ESS	-22.1	-376.6	0.24	0.17	0.23	0.10	0.00	0.00	0.53	0.09	0.00	0.00
YS-20	73.305	139.893	ESS	-26.9	-718.6	0.27	0.17	0.63	0.14	0.00	0.00	0.11	0.07	0.00	0.00
YS-21	73.089	140.348	ESS	-27	-748.8	0.24	0.16	0.66	0.14	0.00	0.00	0.09	0.07	0.00	0.00
YS-23	72.789	142.670	ESS	-27.2	-708.9	0.30	0.19	0.59	0.15	0.00	0.00	0.11	0.08	0.00	0.00
YS-24	73.048	142.667	ESS	-27.4	-687	0.34	0.20	0.54	0.16	0.00	0.00	0.11	0.09	0.00	0.00
YS-25	73.143	142.667	ESS	-27.3	-748.3	0.27	0.18	0.63	0.15	0.00	0.00	0.09	0.07	0.00	0.00
YS-26	72.460	150.596	ESS	-27.4	-740.7	0.26	0.18	0.64	0.15	0.00	0.00	0.09	0.07	0.00	0.00
YS-28	72.651	154.185	ESS	-26.2	-672.4	0.27	0.17	0.59	0.13	0.00	0.00	0.14	0.09	0.00	0.00
YS-29	72.200	153.166	ESS	-26.6	-675.9	0.28	0.17	0.60	0.13	0.00	0.00	0.13	0.09	0.00	0.00
YS-30	71.358	152.153	ESS	-27.4	-681.8	0.31	0.19	0.58	0.15	0.00	0.00	0.12	0.09	0.00	0.00
YS-104	76.934	155.169	ESS	-23.1	-464.6	0.28	0.22	0.32	0.13	0.00	0.00	0.39	0.11	0.00	0.00
YS-106	76.969	150.291	ESS	-23.8	-515.5	0.32	0.23	0.36	0.14	0.00	0.00	0.32	0.11	0.00	0.00
YS-112	74.833	159.330	ESS	-24.6	-606.6	0.29	0.22	0.47	0.15	0.00	0.00	0.23	0.10	0.00	0.00
YS-116	74.583	157.003	ESS	-25.5	-681.8	0.27	0.18	0.57	0.13	0.00	0.00	0.16	0.08	0.00	0.00
YS-120	73.292	155.168	ESS	-25	-600.3	0.28	0.20	0.50	0.13	0.00	0.00	0.22	0.10	0.00	0.00
SW-40	77.681	144.690	ESS	-23.7	-456.7	0.33	0.23	0.29	0.13	0.00	0.00	0.38	0.12	0.00	0.00
SW-41	77.320	147.830	ESS	-21.4	-469	0.19	0.18	0.36	0.12	0.00	0.00	0.45	0.08	0.00	0.00
SW-43	76.780	147.791	ESS	-24.6	-447.1	0.38	0.23	0.28	0.13	0.00	0.00	0.34	0.13	0.00	0.00
SW-44	76.273	146.034	ESS	-24.8	-478.6	0.38	0.23	0.31	0.13	0.00	0.00	0.31	0.13	0.00	0.00
SW-46	76.403	149.878	ESS	-24.7	-459.8	0.38	0.23	0.30	0.13	0.00	0.00	0.32	0.13	0.00	0.00
SW-48	76.616	153.365	ESS	-25.8	-345.3	0.43	0.23	0.18	0.10	0.00	0.00	0.39	0.17	0.00	0.00
SW-49	76.526	156.924	ESS	-23.6	-367.9	0.32	0.21	0.23	0.11	0.00	0.00	0.45	0.13	0.00	0.00

SW-50	75.763	158.529	ESS	-24.6	-514.7	0.36	0.23	0.35	0.14	0.00	0.00	0.29	0.12	0.00	0.00
SW-55	74.845	159.327	ESS	-25.1	-326.3	0.41	0.23	0.18	0.10	0.00	0.00	0.41	0.17	0.00	0.00
lv57-12	72.567	158.501	ESS	-25.5	-536.5	0.36	0.21	0.41	0.13	0.00	0.00	0.23	0.12	0.00	0.00
lv57-14	73.690	149.597	ESS	-24.8	-595	0.28	0.20	0.49	0.13	0.00	0.00	0.23	0.10	0.00	0.00
lv57-15	74.400	144.827	ESS	-25.1	-567.6	0.33	0.22	0.44	0.14	0.00	0.00	0.23	0.11	0.00	0.00
lv57-88	73.030	148.181	ESS	-26.4	-718.3	0.24	0.16	0.64	0.13	0.00	0.00	0.11	0.07	0.00	0.00
lv57-90	71.618	158.282	ESS	-26.3	-681.3	0.28	0.17	0.59	0.13	0.00	0.00	0.13	0.08	0.00	0.00
IK43-46	72.100	150.767	ESS	-26.6	-527.3	0.38	0.20	0.43	0.13	0.00	0.00	0.19	0.13	0.00	0.00
25-MC6	79.226	152.676	ESS	-22.4	-467.2	0.26	0.21	0.32	0.13	0.00	0.00	0.42	0.09	0.00	0.00
N?68	72.567	150.936	ESS	-25.9	-804.5	0.15	0.12	0.76	0.10	0.00	0.00	0.09	0.05	0.00	0.00
YS-4	75.987	129.984	LS	-24.3	-436.6	0.35	0.23	0.29	0.12	0.00	0.00	0.35	0.14	0.00	0.00
YS-6	74.724	130.016	LS	-25.7	-465.2	0.41	0.22	0.33	0.13	0.00	0.00	0.26	0.14	0.00	0.00
YS-9	73.366	129.997	LS	-26.1	-422.7	0.43	0.22	0.31	0.12	0.00	0.00	0.26	0.15	0.00	0.00
YS-13	71.968	131.701	LS	-25.9	-542.6	0.34	0.20	0.45	0.13	0.00	0.00	0.21	0.12	0.00	0.00
YS-14	71.630	130.050	LS	-26.2	-504.4	0.37	0.20	0.41	0.13	0.00	0.00	0.21	0.13	0.00	0.00
YS-19	73.035	133.456	LS	-25.8	-556.8	0.34	0.20	0.45	0.13	0.00	0.00	0.21	0.12	0.00	0.00
YS-22	72.875	140.629	LS	-27.4	-716.2	0.30	0.19	0.59	0.16	0.00	0.00	0.10	0.08	0.00	0.00
YS-131	76.398	125.473	LS	-24.3	-433.1	0.35	0.23	0.29	0.12	0.00	0.00	0.36	0.14	0.00	0.00
TB-17	72.291	132.921	LS	-25.6	-506.4	0.36	0.21	0.40	0.13	0.00	0.00	0.24	0.13	0.00	0.00
TB-19	72.086	132.776	LS	-26	-621.6	0.29	0.18	0.54	0.13	0.00	0.00	0.17	0.10	0.00	0.00
TB-24	71.764	131.166	LS	-25.6	-549.3	0.32	0.19	0.46	0.13	0.00	0.00	0.22	0.12	0.00	0.00
TB-28	71.618	130.037	LS	-26.2	-513.3	0.37	0.20	0.42	0.13	0.00	0.00	0.21	0.13	0.00	0.00
TB-34	72.287	131.088	LS	-25.7	-526.3	0.34	0.20	0.44	0.13	0.00	0.00	0.22	0.12	0.00	0.00
TB-38	72.925	130.842	LS	-26	-481.7	0.39	0.21	0.38	0.13	0.00	0.00	0.23	0.14	0.00	0.00
TB-43	72.892	131.931	LS	-25.6	-517.1	0.35	0.20	0.41	0.13	0.00	0.00	0.23	0.13	0.00	0.00
TB-46	72.701	130.175	LS	-26.5	-436	0.43	0.22	0.33	0.12	0.00	0.00	0.24	0.15	0.00	0.00
TB-51	72.457	131.657	LS	-25.8	-495.2	0.37	0.21	0.40	0.13	0.00	0.00	0.23	0.13	0.00	0.00
TB-59	72.092	130.063	LS	-26	-492.6	0.37	0.20	0.40	0.13	0.00	0.00	0.22	0.13	0.00	0.00
SW-6	77.150	127.352	LS	-23.2	-363.9	0.30	0.21	0.24	0.11	0.00	0.00	0.47	0.13	0.00	0.00
SW-13	76.777	125.830	LS	-24.1	-325.5	0.35	0.22	0.19	0.10	0.00	0.00	0.45	0.15	0.00	0.00
SW-14	76.894	127.798	LS	-24.3	-314	0.36	0.22	0.18	0.09	0.00	0.00	0.46	0.16	0.00	0.00
SW-18	76.399	125.460	LS	-24.9	-289.2	0.39	0.23	0.17	0.09	0.00	0.00	0.44	0.18	0.00	0.00
SW-19	76.456	126.211	LS	-23.9	-295.2	0.34	0.21	0.18	0.09	0.00	0.00	0.49	0.16	0.00	0.00
SW-20	76.454	126.742	LS	-23.8	-317.1	0.34	0.21	0.19	0.09	0.00	0.00	0.47	0.15	0.00	0.00
SW-21	76.126	127.190	LS	-24.2	-320.7	0.36	0.22	0.19	0.10	0.00	0.00	0.45	0.16	0.00	0.00
SW-23	76.171	129.333	LS	-25	-333.3	0.41	0.23	0.19	0.10	0.00	0.00	0.40	0.17	0.00	0.00
SW-24	75.599	129.558	LS	-24.3	-284.3	0.36	0.22	0.17	0.09	0.00	0.00	0.47	0.17	0.00	0.00
SW-26	76.473	132.044	LS	-24.4	-440.8	0.37	0.23	0.29	0.13	0.00	0.00	0.35	0.14	0.00	0.00
SW-28	77.342	135.007	LS	-23.8	-416.5	0.33	0.22	0.26	0.12	0.00	0.00	0.40	0.13	0.00	0.00
SW-29	77.753	136.545	LS	-23.4	-452.5	0.31	0.22	0.30	0.13	0.00	0.00	0.39	0.12	0.00	0.00
SW-30	78.182	138.355	LS	-23.3	-465.1	0.31	0.22	0.31	0.13	0.00	0.00	0.39	0.11	0.00	0.00
lv78-25	75.408	134.741	LS	-24.9	-496.9	0.36	0.23	0.35	0.13	0.00	0.00	0.29	0.13	0.00	0.00
lv78-27	74.764	135.213	LS	-24.8	-531.5	0.33	0.22	0.40	0.14	0.00	0.00	0.27	0.12	0.00	0.00
lv78-29	74.138	137.105	LS	-25	-717.2	0.21	0.17	0.64	0.13	0.00	0.00	0.16	0.08	0.00	0.00
lv78-31	73.548	138.924	LS	-25.1	-678.7	0.23	0.18	0.60	0.13	0.00	0.00	0.17	0.08	0.00	0.00

lv78-40	72.883	137.619	LS	-25.6	-531.3	0.35	0.20	0.43	0.13	0.00	0.00	0.22	0.12	0.00	0.00
N-8	72.600	129.900	LS	-25	-608.3	0.25	0.18	0.54	0.12	0.00	0.00	0.22	0.10	0.00	0.00
YS-11	71.960	129.540	LS	-25	-609	0.24	0.18	0.54	0.12	0.00	0.00	0.21	0.10	0.00	0.00
N?24	74.750	114.009	LS	-23.8	-526.5	0.26	0.21	0.42	0.13	0.00	0.00	0.32	0.11	0.00	0.00
N?39	72.573	132.819	LS	-25.3	-551.9	0.32	0.20	0.46	0.13	0.00	0.00	0.23	0.12	0.00	0.00
Ob-1	73.083	73.733	KS	-27.4	-313.8	0.41	0.24	0.00	0.00	0.34	0.19	0.25	0.15	0.00	0.00
Ye-2	73.017	80.433	KS	-26.5	-174.9	0.39	0.23	0.00	0.00	0.25	0.16	0.36	0.21	0.00	0.00
YS-2	73.410	73.000	KS	-27.4	-220	0.40	0.24	0.00	0.00	0.22	0.14	0.38	0.20	0.00	0.00
YS-3	73.490	79.890	KS	-26.9	-216	0.40	0.24	0.00	0.00	0.28	0.17	0.32	0.19	0.00	0.00
N?10	73.843	72.701	KS	-25.7	-278.9	0.38	0.23	0.00	0.00	0.28	0.16	0.34	0.16	0.00	0.00
N?16	73.005	78.373	KS	-26.2	-365.2	0.37	0.23	0.00	0.00	0.44	0.21	0.20	0.12	0.00	0.00
PSh128-11	75.385	64.302	KS	-23.3	-391.6	0.31	0.22	0.00	0.00	0.34	0.18	0.35	0.11	0.00	0.00
KAR94-7	74.330	84.290	KS	-25.6	-274.1	0.38	0.22	0.00	0.00	0.31	0.17	0.32	0.16	0.00	0.00
KAR94-10	76.998	85.252	KS	-23.5	-362.7	0.31	0.21	0.00	0.00	0.32	0.17	0.37	0.11	0.00	0.00
KAR94-32	78.254	86.810	KS	-23.3	-271	0.27	0.18	0.00	0.00	0.21	0.12	0.52	0.11	0.00	0.00
KAR94-36	76.477	73.411	KS	-24	-239.5	0.29	0.19	0.00	0.00	0.20	0.12	0.51	0.14	0.00	0.00
KAR94-39	74.994	72.196	KS	-24	-166.9	0.26	0.19	0.00	0.00	0.15	0.10	0.59	0.15	0.00	0.00
KAR94-41	76.163	76.603	KS	-24.5	-494.4	0.34	0.24	0.00	0.00	0.47	0.22	0.18	0.10	0.00	0.00
KAR94-44	75.765	80.403	KS	-24	-179.4	0.28	0.19	0.00	0.00	0.17	0.11	0.55	0.16	0.00	0.00
KAR94-54	72.668	73.967	KS	-28.2	-230.3	0.41	0.26	0.00	0.00	0.25	0.16	0.34	0.20	0.00	0.00
KAR94-58	71.503	72.581	KS	-28.7	-179.2	0.38	0.26	0.00	0.00	0.23	0.16	0.40	0.23	0.00	0.00
KAR94-61	70.340	73.438	KS	-28.6	-173.5	0.38	0.26	0.00	0.00	0.27	0.19	0.35	0.23	0.00	0.00
KAR94-74	73.342	74.987	KS	-24.5	-93.7	0.28	0.21	0.00	0.00	0.12	0.09	0.60	0.21	0.00	0.00
KAR94-83	72.100	82.002	KS	-27.1	-201.1	0.39	0.23	0.00	0.00	0.32	0.19	0.29	0.19	0.00	0.00
KAR94-85	72.434	80.020	KS	-27.2	-182.2	0.40	0.24	0.00	0.00	0.27	0.17	0.33	0.21	0.00	0.00
KAR94-91	73.550	80.039	KS	-26.4	-143.2	0.38	0.24	0.00	0.00	0.19	0.13	0.42	0.23	0.00	0.00
KAR94-96	73.842	74.563	KS	-26	-178.6	0.37	0.23	0.00	0.00	0.19	0.12	0.44	0.20	0.00	0.00
KAR94-100	73.835	70.268	KS	-25.9	-93.5	0.28	0.23	0.00	0.00	0.10	0.07	0.62	0.23	0.00	0.00
KAR94-104	75.001	68.821	KS	-24.5	-219	0.30	0.20	0.00	0.00	0.19	0.11	0.52	0.15	0.00	0.00
KAR94-107	74.000	65.006	KS	-22.7	-231	0.24	0.17	0.00	0.00	0.20	0.12	0.55	0.11	0.00	0.00
KAR94-109	73.000	60.535	KS	-23.3	-288.5	0.28	0.19	0.00	0.00	0.25	0.14	0.46	0.12	0.00	0.00
KAR94-110	71.999	58.795	KS	-22.3	-324.6	0.25	0.19	0.00	0.00	0.30	0.16	0.44	0.10	0.00	0.00
KAR94-111	70.999	58.334	KS	-23.4	-343.8	0.30	0.22	0.00	0.00	0.35	0.18	0.36	0.12	0.00	0.00
PSh55-4930	65.663	36.421	BS	-24.3	-146.1	0.38	0.23	0.00	0.00	0.00	0.00	0.55	0.23	0.07	0.05
PSh55-4944	65.550	38.000	BS	-24.8	-49.7	0.53	0.24	0.00	0.00	0.00	0.00	0.42	0.25	0.05	0.05
PSh55-4913	65.803	39.253	BS	-24.8	-108.8	0.44	0.25	0.00	0.00	0.00	0.00	0.49	0.26	0.07	0.05
PSh55-4921	65.084	39.741	BS	-27.3	-151.6	0.65	0.23	0.00	0.00	0.00	0.00	0.25	0.23	0.11	0.08
PSh55-4932	66.334	34.657	BS	-24.5	-55.7	0.44	0.27	0.00	0.00	0.00	0.00	0.53	0.29	0.03	0.04
ASV13-1106	69.152	57.913	BS	-24.1	-307.2	0.38	0.21	0.00	0.00	0.00	0.00	0.44	0.17	0.18	0.08
ASV13-1121	69.590	58.693	BS	-24.5	-309.3	0.42	0.21	0.00	0.00	0.00	0.00	0.41	0.18	0.17	0.08
ASV13-1132	70.380	55.562	BS	-23	-251.1	0.30	0.18	0.00	0.00	0.00	0.00	0.56	0.15	0.14	0.06
ASV13-1136	69.683	54.583	BS	-23.9	-307.1	0.37	0.21	0.00	0.00	0.00	0.00	0.45	0.17	0.18	0.08
ASV13-1140	69.167	54.318	BS	-23.6	-246.7	0.35	0.20	0.00	0.00	0.00	0.00	0.51	0.18	0.15	0.07
ASV13-1143	68.783	52.327	BS	-22.7	-173.1	0.27	0.18	0.00	0.00	0.00	0.00	0.63	0.17	0.09	0.05
ASV13-1148	68.636	47.746	BS	-22.6	-221.1	0.27	0.17	0.00	0.00	0.00	0.00	0.62	0.15	0.11	0.05

ASV13-1152	69.533	50.650	BS	-23.1	-196.7	0.30	0.19	0.00	0.00	0.00	0.00	0.60	0.17	0.10	0.05
ASV13-1153	69.917	51.683	BS	-22.7	-203.1	0.27	0.17	0.00	0.00	0.00	0.00	0.62	0.16	0.10	0.05
ASV13-1156	70.550	52.800	BS	-22.4	-188.4	0.25	0.16	0.00	0.00	0.00	0.00	0.66	0.14	0.09	0.05
ASV13-1163	68.633	50.140	BS	-24	-279.9	0.38	0.21	0.00	0.00	0.00	0.00	0.46	0.18	0.16	0.08
NZ94-2	69.458	39.018	BS	-21.6	-130.4	0.14	0.11	0.00	0.00	0.00	0.00	0.81	0.10	0.05	0.03
NZ94-5	69.950	40.895	BS	-21.7	-63.1	0.06	0.09	0.00	0.00	0.00	0.00	0.92	0.10	0.01	0.01
NZ94-7	70.493	42.413	BS	-21.9	-175.9	0.19	0.13	0.00	0.00	0.00	0.00	0.74	0.11	0.07	0.04
NZ94-9	71.521	46.628	BS	-22	-210.4	0.22	0.14	0.00	0.00	0.00	0.00	0.68	0.12	0.10	0.05
NZ94-10	70.711	48.346	BS	-22.4	-159.8	0.22	0.16	0.00	0.00	0.00	0.00	0.71	0.14	0.07	0.04
NZ94-11	70.182	48.806	BS	-23	-167.9	0.27	0.18	0.00	0.00	0.00	0.00	0.66	0.17	0.07	0.04
NZ94-21	68.823	44.697	BS	-23.3	-93.2	0.21	0.20	0.00	0.00	0.00	0.00	0.76	0.20	0.03	0.02
NHT	80.397	16.200	BS	-23.4	-337.1	0.31	0.19	0.00	0.00	0.00	0.00	0.46	0.15	0.23	0.08
CAB-8	81.278	26.863	BS	-22.8	-475.3	0.22	0.17	0.00	0.00	0.00	0.00	0.41	0.11	0.37	0.09
CAB-11	79.942	30.280	BS	-24.1	-393.3	0.35	0.20	0.00	0.00	0.00	0.00	0.38	0.15	0.28	0.10
EE4	79.391	29.006	BS	-22.8	-343.5	0.26	0.18	0.00	0.00	0.00	0.00	0.50	0.13	0.24	0.08
EE2	78.913	22.804	BS	-25.1	-418.7	0.42	0.21	0.00	0.00	0.00	0.00	0.29	0.16	0.29	0.11
1/G20	74.850	33.220	BS	-23	-355.8	0.30	0.19	0.00	0.00	0.00	0.00	0.49	0.13	0.21	0.09
3/G16	75.150	28.580	BS	-22.4	-504	0.19	0.16	0.00	0.00	0.00	0.00	0.41	0.09	0.40	0.09
11/G14	75.368	26.620	BS	-22.6	-379.8	0.25	0.18	0.00	0.00	0.00	0.00	0.49	0.12	0.26	0.09
32/G12	75.922	25.330	BS	-23.7	-371.8	0.33	0.20	0.00	0.00	0.00	0.00	0.42	0.15	0.24	0.09
PKF #853 C	78.568	10.122	BS	-22.6	-245.5	0.27	0.17	0.00	0.00	0.00	0.00	0.58	0.15	0.15	0.06
SR 1 #925	75.842	16.626	BS	-23.5	-493.9	0.26	0.19	0.00	0.00	0.00	0.00	0.35	0.12	0.39	0.10
SW-1	78.950	125.232	CAO	-22.3	-418.1	0.24	0.18	0.27	0.11	0.00	0.00	0.49	0.09	0.00	0.00
SW-2	78.581	125.607	CAO	-22.7	-391.8	0.27	0.19	0.24	0.10	0.00	0.00	0.49	0.11	0.00	0.00
SW-3	78.238	126.150	CAO	-22.6	-426.3	0.25	0.19	0.28	0.11	0.00	0.00	0.47	0.10	0.00	0.00
SW-4	77.938	126.518	CAO	-22.5	-428	0.24	0.19	0.29	0.11	0.00	0.00	0.47	0.10	0.00	0.00
SW-31	79.396	135.497	CAO	-23.1	-489.4	0.28	0.21	0.32	0.13	0.00	0.00	0.40	0.10	0.00	0.00
SW-32	79.093	135.760	CAO	-22.8	-455.3	0.27	0.20	0.29	0.12	0.00	0.00	0.44	0.10	0.00	0.00
SW-33	78.927	136.178	CAO	-22.8	-425.6	0.27	0.19	0.26	0.11	0.00	0.00	0.46	0.10	0.00	0.00
SW-34	78.758	136.501	CAO	-22.8	-418.5	0.28	0.19	0.25	0.11	0.00	0.00	0.47	0.10	0.00	0.00
SW-35	78.600	137.061	CAO	-23.3	-430.2	0.30	0.20	0.26	0.11	0.00	0.00	0.44	0.11	0.00	0.00
SW-67	76.333	175.579	CAO	-21.2	-468.1	0.19	0.17	0.33	0.12	0.00	0.00	0.48	0.07	0.00	0.00
27-MC6	79.665	154.126	CAO	-22.6	-454.8	0.26	0.19	0.29	0.12	0.00	0.00	0.45	0.09	0.00	0.00
PS87/023-2	86.631	-44.874	CAO	-22.5	-386.6	0.26	0.17	0.21	0.10	0.00	0.00	0.53	0.08	0.00	0.00
PS87/030-2	88.659	-61.487	CAO	-22.5	-637.6	0.27	0.27	0.44	0.19	0.00	0.00	0.29	0.09	0.00	0.00
PS87/055-1	85.691	148.991	CAO	-22.5	-343	0.22	0.13	0.16	0.09	0.00	0.00	0.61	0.05	0.00	0.00
PS87/070-3	83.803	146.117	CAO	-22.3	-225.5	0.15	0.09	0.09	0.06	0.00	0.00	0.75	0.04	0.00	0.00
PS87/079-3	83.202	141.376	CAO	-22.3	-85.9	0.04	0.02	0.02	0.01	0.00	0.00	0.94	0.02	0.00	0.00
PS87/099-4	81.425	142.239	CAO	-22.7	-76.1	0.03	0.02	0.02	0.01	0.00	0.00	0.95	0.02	0.00	0.00

*The abbreviations of the shelf seas are CAA – Canadian Arctic Archipelago, BFS – Beaufort Sea, CS – Chukchi Sea, ESS – East Siberian Sea, LS – Laptev Sea, KS – Kara Sea, BS – Barents Sea, CAO – Central Arctic Ocean.

Supplementary Table 6: Collection of sediment mass accumulation rates (MAR) from the circum-Arctic shelves

Sample ID	Lat	Lon	Shelf Sea	Citation	MAR g cm^{-2} yr^{-1}	Conversion*
28-MC/I	77.342	135.007	Laptev Sea	This study	0.113	
40-MC/III	77.681	144.690	East Siberian Sea	This study	0.161	
48-MC/I	76.616	153.365	East Siberian Sea	This study	0.096	
YS-19 2B	73.035	133.456	Laptev Sea	This study	0.252	
YS-2 3A	73.410	73.000	Kara Sea	This study	0.136	
YS-3 4A	73.490	79.890	Kara Sea	This study	0.087	
YS-40 2B	71.483	170.553	East Siberian Sea	This study	0.321	
SW-14	76.894	127.798	Laptev Sea	This study	0.092	
SW-23	76.171	129.333	Laptev Sea	This study	0.099	
SW-24	75.599	129.558	Laptev Sea	This study	0.195	
SW-58	74.439	166.047	East Siberian Sea	This study	0.094	
YS-6	74.724	130.016	Laptev Sea	This study	0.284	
I	75.667	30.167	Barents Sea	Carroll et al. 2008	0.033	from OC flux
II	78.250	27.167	Barents Sea	Carroll et al. 2008	0.035	from OC flux
III	79.017	25.767	Barents Sea	Carroll et al. 2008	0.051	from OC flux
IV	77.017	29.483	Barents Sea	Carroll et al. 2008	0.031	from OC flux
X	79.367	28.700	Barents Sea	Carroll et al. 2008	0.063	from OC flux
XI	79.950	30.283	Barents Sea	Carroll et al. 2008	0.039	from OC flux
XII	80.150	29.600	Barents Sea	Carroll et al. 2008	0.032	from OC flux
XVI	77.083	28.550	Barents Sea	Carroll et al. 2008	0.063	from OC flux
XVII	77.433	40.300	Barents Sea	Carroll et al. 2008	0.036	from OC flux
XVIII	75.683	31.817	Barents Sea	Carroll et al. 2008	0.029	from OC flux
H1	76.968	15.729	Barents Sea	Koziorowska et al. 2018	0.131	
H2	76.940	15.316	Barents Sea	Koziorowska et al. 2018	0.233	
Kb1	79.026	11.474	Barents Sea	Koziorowska et al. 2018	0.116	
Kb2	78.948	11.965	Barents Sea	Koziorowska et al. 2018	0.195	
3	66.333	33.667	Barents Sea	Lisitzin et al. 2015	0.015	
4	65.167	37.933	Barents Sea	Lisitzin et al. 2015	0.051	
32	64.117	37.583	Barents Sea	Lisitzin et al. 2015	0.081	
44	64.967	39.517	Barents Sea	Lisitzin et al. 2015	0.078	
46	65.100	39.283	Barents Sea	Lisitzin et al. 2015	0.033	
59	66.333	35.533	Barents Sea	Lisitzin et al. 2015	0.019	
66	65.033	34.883	Barents Sea	Lisitzin et al. 2015	0.025	
76	65.283	39.267	Barents Sea	Lisitzin et al. 2015	0.027	
77	65.133	39.283	Barents Sea	Lisitzin et al. 2015	0.009	
78	65.083	39.733	Barents Sea	Lisitzin et al. 2015	0.126	
4697	65.283	38.917	Barents Sea	Lisitzin et al. 2015	0.012	
4698	65.417	38.667	Barents Sea	Lisitzin et al. 2015	0.024	
4706	65.083	36.100	Barents Sea	Lisitzin et al. 2015	0.026	
4720	65.950	35.883	Barents Sea	Lisitzin et al. 2015	0.066	

4943	65.833	37.500	Barents Sea	Lisitzin et al. 2015	0.021	
MIC-36	74.967	47.000	Barents Sea	Maiti et al. 2010	0.053	from OC flux
MIC-4	75.033	26.217	Barents Sea	Maiti et al. 2010	0.054	from OC flux
PIC-8	77.983	26.833	Barents Sea	Maiti et al. 2010	0.060	from OC flux
POW−1	73.133	25.633	Barents Sea	Maiti et al. 2010	0.034	from OC flux
POW-17	73.050	35.583	Barents Sea	Maiti et al. 2010	0.046	from OC flux
PIC-40	78.233	53.117	Barents Sea	Maiti et al. 2010	0.084	from OC flux
6	69.639	50.753	Barents Sea	Smith et al. 1995	0.127	from LSR
8	70.516	54.643	Barents Sea	Smith et al. 1995	0.095	from LSR
12	70.283	55.607	Barents Sea	Smith et al. 1995	0.215	from LSR
BASICC 1	73.097	25.633	Barents Sea	Vare et al. 2010	0.087	from LSR
BASICC 8	77.983	26.794	Barents Sea	Vare et al. 2010	0.087	from LSR
BASICC 43	72.539	45.738	Barents Sea	Vare et al. 2010	0.103	from LSR
803BC	70.636	-135.917	Beaufort Sea	Bringué & Rochon 2012	0.092	from LSR
SL8979-1	70.533	-150.117	Beaufort Sea	Naidu et al. 1999	2.050	
SL8979-2	70.517	-150.017	Beaufort Sea	Naidu et al. 1999	1.030	
SL8979-3	70.517	-149.950	Beaufort Sea	Naidu et al. 1999	0.580	
SL8979-5	70.533	-149.750	Beaufort Sea	Naidu et al. 1999	0.730	
SL8979-6	70.533	-149.667	Beaufort Sea	Naidu et al. 1999	0.930	
SL8979-7	70.533	-149.583	Beaufort Sea	Naidu et al. 1999	0.650	
2004-804-912A	69.488	-137.941	Beaufort Sea	Richerol et al. 2008	0.032	from LSR
2004-804-909B	69.753	-138.272	Beaufort Sea	Richerol et al. 2008	0.067	from LSR
2004-804-906B	70.019	-138.597	Beaufort Sea	Richerol et al. 2008	0.095	from LSR
L1 (SL2)	70.525	-150.202	Beaufort Sea	Schreiner et al. 2013	0.366	from LSR
L2 (SL4)	70.496	-150.061	Beaufort Sea	Schreiner et al. 2013	0.056	from LSR
L3 (SL1)	70.531	-149.948	Beaufort Sea	Schreiner et al. 2013	0.294	from LSR
L4 (SL3)	70.537	-149.882	Beaufort Sea	Schreiner et al. 2013	0.032	from LSR
L6 (SL6)	70.524	-149.379	Beaufort Sea	Schreiner et al. 2013	0.191	from LSR
MA680BC	69.604	-138.226	Beaufort Sea	Durantou et al. 2012	0.254	from LSR
QM1	68.667	-103.004	CAA	Kuzyk et al. 2017	0.110	
FS1	70.999	-97.998	CAA	Kuzyk et al. 2017	0.090	
VS1	70.249	-98.929	CAA	Kuzyk et al. 2017	0.040	
PS1	73.597	-96.295	CAA	Kuzyk et al. 2017	0.060	
PS2	73.000	-96.218	CAA	Kuzyk et al. 2017	0.210	
CAA2	73.935	-86.108	CAA	Kuzyk et al. 2017	0.130	
BE2	71.973	-95.998	CAA	Kuzyk et al. 2017	0.110	
CAA1	73.927	-81.814	CAA	Kuzyk et al. 2017	0.180	
BB11	73.933	-77.930	CAA	Kuzyk et al. 2017	0.050	
99LLSL-001E	68.095	-114.186	CAA	Pieńkowski et al. 2017	0.102	from OC flux
99LSSL-001	68.095	-114.186	CAA	Pieńkowski et al. 2017	0.105	from OC flux
535-BC	71.077	-139.658	CAA	Letaief et al. 2021	0.070	from LSR
408-BC	71.304	-127.575	CAA	Letaief et al. 2021	0.069	from LSR
05-BC	67.865	-115.072	CAA	Letaief et al. 2021	0.142	from LSR
316-BC	68.389	-112.092	CAA	Letaief et al. 2021	0.088	from LSR
QMG4-BC	68.490	-103.419	CAA	Letaief et al. 2021	0.130	from LSR

304-BC	74.246	-91.522	CAA	Letaief et al. 2021	0.146	from LSR
165-BC	72.709	-75.761	CAA	Letaief et al. 2021	0.065	from LSR
ARC-4	69.166	-100.695	CAA	Belt et al. 2010	0.127	from LSR
ARC-5	68.991	-106.571	CAA	Belt et al. 2010	0.064	from LSR
2004-804-009	74.187	-81.195	CAA	Ledu et al. 2008	0.048	from LSR
2004-804-004	74.268	-91.073	CAA	Ledu et al. 2010	0.027	from LSR
VIII	81.283	26.850	CAO	Carroll et al. 2008	0.057	from OC flux
6	75.350	-170.500	CAO	Huh et al. 1997	0.055	
BC3	71.578	-156.018	CAO	Kuzyk et al. 2017	0.160	
CG1	70.562	-142.858	CAO	Kuzyk et al. 2017	0.180	
SS3	70.973	-134.694	CAO	Kuzyk et al. 2017	0.040	
CG3	70.461	-140.160	CAO	Kuzyk et al. 2017	0.070	
BC4	71.930	-154.887	CAO	Kuzyk et al. 2017	0.160	
CG2	70.700	-142.833	CAO	Kuzyk et al. 2017	0.070	
L50	70.413	-139.090	CAO	Kuzyk et al. 2017	0.060	
BC5	72.000	-154.708	CAO	Kuzyk et al. 2017	0.050	
BC6	72.233	-154.037	CAO	Kuzyk et al. 2017	0.080	
CH-21	71.203	-164.200	Chukchi Sea	Baskaran et al. 1995	0.115	
KS-1	66.617	-163.005	Chukchi Sea	Baskaran et al. 1995	0.010	
SU-11	67.038	-165.745	Chukchi Sea	Baskaran et al. 1995	0.031	
SU-84-12	71.483	-165.117	Chukchi Sea	Baskaran et al. 1995	0.324	
CH-40	70.278	-167.905	Chukchi Sea	Baskaran et al. 1995	0.119	
CH-13	72.518	-164.133	Chukchi Sea	Baskaran et al. 1995	0.167	
CH-39	71.870	-168.257	Chukchi Sea	Baskaran et al. 1995	0.090	
SU-5	67.033	-169.000	Chukchi Sea	Baskaran et al. 1995	0.159	
CH-25	72.627	-167.075	Chukchi Sea	Baskaran et al. 1995	0.083	
CH-38	70.700	-167.382	Chukchi Sea	Baskaran et al. 1995	0.156	
CH21	71.203	-164.200	Chukchi Sea	Feder et al. 1994	0.215	from OC flux
CH26	71.533	-167.785	Chukchi Sea	Feder et al. 1994	0.214	from OC flux
CH40	70.278	-167.905	Chukchi Sea	Feder et al. 1994	0.215	from OC flux
UTN3	67.334	-169.000	Chukchi Sea	Kuzyk et al. 2017	0.280	
UTN5	67.670	-168.958	Chukchi Sea	Kuzyk et al. 2017	0.280	
UTN7	68.000	-168.933	Chukchi Sea	Kuzyk et al. 2017	0.280	
b16	72.544	-175.995	Chukchi Sea	Vologina et al. 2016	0.072	from LSR
ARC6-14R07	72.996	-168.970	Chukchi Sea	Astakhov et al. 2019	0.067	from LSR
ARC6-14R08	74.008	-168.975	Chukchi Sea	Astakhov et al. 2019	0.072	from LSR
ARC6-14R09	74.612	-168.986	Chukchi Sea	Astakhov et al. 2019	0.035	from LSR
ARC6-14S03	72.235	-157.053	Chukchi Sea	Astakhov et al. 2019	0.127	from LSR
LV77-1	67.700	-173.000	Chukchi Sea	Li et al. 2020	0.133	from porosity
LV77-5	69.711	-173.200	Chukchi Sea	Li et al. 2020	0.154	from porosity
14R07	73.001	-167.964	Chukchi Sea	Li et al. 2020	0.056	from porosity
HLY0501	72.900	158.420	Chukchi Sea	Deschamps et al. 2017	0.052	from LSR
IK-105-A	70.484	173.130	East Siberian Sea	Vonk et al. 2012	0.286	
IK-110-A	70.834	162.877	East Siberian Sea	Vonk et al. 2012	0.162	
IK-114-A	75.000	153.526	East Siberian Sea	Vonk et al. 2012	0.290	

YS-120	73.292	155.168	East Siberian Sea	Vonk et al. 2012	0.203	
YS-22	72.875	140.629	East Siberian Sea	Vonk et al. 2012	0.579	
YS-26	72.460	150.596	East Siberian Sea	Vonk et al. 2012	0.630	
YS-35	69.817	164.057	East Siberian Sea	Vonk et al. 2012	0.275	
YS-36	69.817	165.999	East Siberian Sea	Vonk et al. 2012	0.211	
YS-37	70.135	168.007	East Siberian Sea	Vonk et al. 2012	0.232	
YS-90	74.668	172.388	East Siberian Sea	Vonk et al. 2012	0.138	
YS-93	74.419	165.999	East Siberian Sea	Vonk et al. 2012	0.195	
YS-98	75.551	160.751	East Siberian Sea	Vonk et al. 2012	0.170	
LV77-12	70.729	174.355	East Siberian Sea	Li et al. 2020	0.278	from porosity
LV77-14	72.233	174.784	East Siberian Sea	Li et al. 2020	0.254	from porosity
LV77-21	74.128	167.503	East Siberian Sea	Li et al. 2020	0.068	from porosity
LV77-24	76.600	168.514	East Siberian Sea	Aksentov et al. 2021	0.025	
LV77-33	75.844	159.262	East Siberian Sea	Li et al. 2020	0.078	from porosity
LV77-40	71.898	153.237	East Siberian Sea	Li et al. 2020	0.198	from porosity
LV77-43	73.370	153.173	East Siberian Sea	Li et al. 2020	0.207	from porosity
1	71.500	58.000	Kara Sea	Hamilton et al. 1994	0.057	
4	75.500	68.000	Kara Sea	Hamilton et al. 1994	0.074	
3	74.500	62.000	Kara Sea	Hamilton et al. 1994	0.079	
OB94-13	69.090	76.717	Kara Sea	Kenna & Sayles 2002	0.246	from LSR
5343	72.093	81.482	Kara Sea	Rusakov et al. 2019	0.541	from LSR
5326	72.168	74.287	Kara Sea	Rusakov et al. 2019	0.843	from LSR
5324	71.472	72.558	Kara Sea	Rusakov et al. 2019	0.557	from LSR
5323	71.687	72.952	Kara Sea	Rusakov et al. 2019	0.604	from LSR
5391	74.180	59.170	Kara Sea	Rusakov et al. 2019	0.199	from LSR
5306	76.348	72.180	Kara Sea	Rusakov et al. 2019	0.119	from LSR
5214	76.530	71.370	Kara Sea	Rusakov et al. 2019	0.175	from LSR
5374	74.672	59.935	Kara Sea	Rusakov et al. 2019	0.159	from LSR
5403	70.883	58.295	Kara Sea	Rusakov et al. 2019	0.111	from LSR
5240	79.250	87.620	Kara Sea	Rusakov et al. 2019	0.127	from LSR
5394	72.375	57.877	Kara Sea	Rusakov et al. 2019	0.159	from LSR
5199	72.330	57.850	Kara Sea	Rusakov et al. 2019	0.246	from LSR
5358	75.385	64.313	Kara Sea	Rusakov et al. 2019	0.739	from LSR
IK-118-A	71.867	131.033	Laptev Sea	Vonk et al. 2012	0.333	

*some MAR data were converted from other parameters; i) from the OC accumulation rate (in g OC m^{-2} yr^{-1}), or ii) when ^{210}Pb-based linear sedimentation rates (LSR) were reported by assuming a dry bulk density of 0.8 g cm^{-3} [2]. Data based on ref[2,33–60].

Supplementary Table 7: Radiochronological data for sediment core slices from the ESAS and the Kara Sea. Also shown are the bulk density (BD), the porosity, and the dry bulk density (DBD) when available.

**Depth**	**BD**	**Porosity**	**DBD**	**^{210}Pb**	**^{226}Ra**	**$^{210}Pb_{xs}$**	**$^{210}Pb_{xs}$**
cm	g cm^{-3}		g cm^{-3}	Bq g^{-1}	Bq g^{-1}	Bq g^{-1}	Bq cm^{-2}
				28-MC/I			
0-1	1.27	0.83	0.44	0.071	0.049	0.023	0.010
1-2	1.33	0.80	0.53	0.064	0.058	0.006	0.003
2-3	1.34	0.79	0.55	0.081	0.063	0.019	0.010
3-4	1.38	0.77	0.62	0.072	0.091	0.000	0.000
4-5	1.39	0.76	0.63	0.079	0.065	0.014	0.009
5-6	1.39	0.76	0.63	0.060	0.081	0.000	0.000
6-7	1.39	0.76	0.62	0.057	0.027	0.029	0.018
7-8	1.42	0.75	0.67	0.041	0.023	0.017	0.012
8-9	1.48	0.71	0.78	0.039	0.025	0.014	0.011
9-10	1.54	0.67	0.87	0.036	0.026	0.010	0.009
11-12	1.60	0.64	0.96	0.031	0.027		
13-14	1.61	0.63	0.98	0.028			
17-18	1.63	0.62	1.02	0.025	0.026		
19-20	1.66	0.60	1.06	0.023			
				40-MC/III			
0-1	1.76	0.54	1.22	0.019	0.012	0.009	0.011
1-2	1.80	0.51	1.29	0.013	0.011	0.004	0.005
2-3	1.88	0.46	1.42	0.017	0.008	0.008	0.011
3-4	1.91	0.45	1.46	0.014		0.004	0.006
4-5	1.95	0.43	1.52	0.014	0.008	0.004	0.007
5-6	1.91	0.45	1.46	0.008	0.008	0.000	0.000
6-7	1.91	0.45	1.47	0.012	0.008	0.002	0.003
7-8	1.92	0.44	1.48	0.012		0.002	0.003
8-9	2.01	0.39	1.62	0.009		0.000	0.000
9-10	2.01	0.39	1.63	0.010	0.010	0.000	0.001
				48-MC/I			
0-1	1.33	0.80	0.53	0.050	0.031	0.025	0.013
1-2	1.41	0.75	0.66	0.043		0.017	0.011
2-3	1.46	0.72	0.74	0.044	0.023	0.018	0.013
3-4	1.58	0.65	0.94	0.040		0.014	0.014
4-5	1.63	0.62	1.01	0.032	0.023	0.006	0.006
5-6	1.65	0.60	1.05	0.038	0.025	0.012	0.013
6-7	1.66	0.60	1.05	0.032		0.006	0.006
7-8	1.65	0.60	1.05	0.023	0.028	0.000	0.000
9-10	1.68	0.59	1.09	0.025	0.026	0.000	0.000
11-12	1.76	0.54	1.21			0.000	0.000
13-14	1.78	0.53	1.25			0.000	0.000
15-16	1.77	0.54	1.23	0.024			

YS-40 2B						
0-1	0.57	1.13	0.034	0.017	0.016	0.018
1-2	0.60	1.05	0.033		0.014	0.015
2-3	0.60	1.05	0.038	0.019	0.020	0.021
3-4	0.58	1.11	0.033		0.015	0.016
5-6	0.50	1.33	0.034	0.017	0.016	0.021
7-8	0.47	1.41	0.032		0.014	0.020
9-10	0.48	1.37	0.034	0.022	0.015	0.021
11-12	0.46	1.44	0.027	0.016	0.009	0.012
13-14	0.45	1.46	0.023		0.005	0.007
15-16	0.44	1.49	0.025	0.016	0.007	0.010
17-18	0.44	1.49	0.019		0.001	0.002
19-20	0.45	1.46	0.016		0.000	
YS-19 2B						
0-1	0.68	0.84	0.056	0.022	0.032	0.027
1-2	0.64	0.95	0.051		0.027	0.026
2-3	0.60	1.07	0.043	0.022	0.019	0.020
3-4	0.57	1.15	0.038		0.014	0.017
5-6	0.57	1.15	0.039	0.024	0.015	0.017
7-8	0.56	1.16	0.042		0.018	0.021
9-10	0.56	1.16	0.050	0.023	0.026	0.030
12-13	0.57	1.15	0.042	0.025	0.018	0.021
14-15	0.56	1.16	0.039		0.015	0.018
15-16	0.55	1.18	0.046	0.025	0.022	0.026
17-18	0.56	1.17	0.041		0.017	0.020
19-20	0.54	1.22	0.040	0.027	0.016	0.020
YS-2 3A						
0-1	0.72	0.75	0.054	0.015	0.042	0.031
1-2	0.65	0.93	0.048		0.036	0.033
2-3	0.63	0.99	0.046	0.013	0.034	0.034
3-4	0.62	1.00	0.052	0.014	0.039	0.039
5-6	0.71	0.76	0.041	0.014	0.028	0.022
7-8	0.64	0.94	0.023		0.011	0.010
9-10	0.62	1.02	0.018	0.011	0.006	0.006
11-12	0.59	1.08	0.017	0.011	0.005	0.005
13-14	0.56	1.17	0.014		0.001	0.001
15-16	0.55	1.21	0.014	0.010	0.002	0.002
YS-3 4A						
0-1	0.81	0.49	0.077	0.022	0.059	0.029
1-2	0.77	0.61	0.069		0.050	0.031
2-3	0.72	0.73	0.058	0.022	0.040	0.029

3-4		0.69	0.83	0.065	0.021	0.047	0.039
5-6		0.80	0.53	0.048	0.016	0.030	0.016
7-8		0.70	0.78	0.032	0.015	0.014	0.011
9-10		0.68	0.86	0.025	0.017	0.006	0.005
11-12		0.67	0.88	0.022		0.003	0.003
13-14		0.66	0.91	0.019	0.016	0.001	0.000
15-16		0.70	0.80	0.019		0.001	0.001
SW-14							
0-1	1.52	0.68	0.84	0.077	0.017	0.0597	0.050
1-2	1.59	0.64	0.94	0.065	0.027	0.0381	0.036
2-3	1.63	0.62	1.01	0.041	0.008	0.0330	0.033
3-4	1.63	0.62	1.01	0.051	0.019	0.0319	0.032
4-5	1.62	0.63	0.99	0.047	0.023	0.0245	0.024
5-6	1.61	0.63	0.98	0.030	0.029	0.0010	0.001
6-7	1.65	0.61	1.04	0.028	0.033	0.0000	
7-8	1.66	0.60	1.05	0.029	0.031	0.0000	
8-9	1.67	0.59	1.08	0.021	0.025	0.0000	
9-10	1.69	0.58	1.11				
10-11	1.74	0.55	1.18	0.022	0.026	0.0000	
11-12	1.77	0.53	1.24	0.022	0.033	0.0000	
12-13	1.79	0.52	1.28	0.012	0.028	0.0000	
13-14	1.77	0.54	1.23	0.021	0.032	0.0000	
14-15	1.72	0.56	1.16	0.018	0.038	0.0000	
15-16	1.73	0.56	1.18				
16-17	1.75	0.55	1.20				
17-18	1.76	0.54	1.21				
18-19	1.80	0.52	1.28				
SW-23							
0-1	1.43	0.74	0.70	0.074	0.023	0.0510	0.036
1-2	1.48	0.71	0.78	0.073	0.027	0.0459	0.036
2-3	1.51	0.69	0.82	0.072	0.026	0.0461	0.038
3-4	1.53	0.68	0.85	0.059	0.031	0.0275	0.023
4-5	1.54	0.67	0.87	0.043	0.026	0.0171	0.015
5-6	1.56	0.66	0.90	0.050	0.051	0.0000	0.000
6-7	1.59	0.64	0.94	0.037	0.027	0.0105	0.010
7-8	1.61	0.63	0.97	0.036	0.032	0.0046	0.004
8-9	1.61	0.63	0.98	0.036	0.048	0.0000	0.000
9-10	1.62	0.62	1.00	0.042	0.048	0.0000	
10-11	1.64	0.61	1.03	0.021	0.038	0.0000	
11-12	1.65	0.60	1.05	0.017	0.032	0.0000	
12-13	1.67	0.59	1.08	0.039	0.021	0.0181	
13-14	1.67	0.59	1.08	0.011	0.019	0.0000	

14-15	1.65	0.61	1.04	0.035	0.029	0.0063	
15-16	1.66	0.60	1.06	0.034	0.027	0.0070	
16-17	1.68	0.59	1.09	0.021	0.028	0.0000	
17-18	1.68	0.59	1.09	0.038	0.026	0.0118	
18-19	1.65	0.61	1.04	0.029	0.039	0.0000	
19-20	1.69	0.58	1.10	0.025	0.026	0.0000	
20-21	1.72	0.57	1.15	0.028	0.036	0.0000	
21-22	1.68	0.59	1.10	0.028	0.038	0.0000	
SW-24							
0-1	1.31	0.82	0.49	0.072	0.034	0.0387	0.019
1-2	1.33	0.80	0.53	0.074	0.030	0.0437	0.023
2-3	1.34	0.79	0.55	0.077	0.016	0.0613	0.034
3-4	1.35	0.79	0.56	0.060	0.042	0.0180	0.010
4-5	1.35	0.79	0.57	0.075	0.032	0.0430	0.024
5-6	1.37	0.78	0.60	0.078	0.045	0.0320	0.019
6-7	1.39	0.76	0.63	0.072	0.007	0.0656	0.041
7-8	1.40	0.76	0.64	0.057	0.021	0.0356	0.023
8-9	1.42	0.75	0.67	0.065	0.034	0.0305	0.020
9-10	1.41	0.75	0.67	0.047	0.027	0.0198	0.013
10-11	1.41	0.75	0.67	0.055	0.030	0.0245	0.016
11-12	1.44	0.73	0.71	0.032	0.035	0.0000	
12-13	1.44	0.73	0.71	0.038	0.049	0.0000	
13-14	1.44	0.73	0.71	0.035	0.043	0.0000	
14-15	1.46	0.72	0.74	0.031	0.041	0.0000	
15-16	1.48	0.71	0.77	0.043	0.026	0.0166	
16-17	1.50	0.70	0.81	0.035	0.034	0.0005	
YS-6							
0-1		0.68	0.86	0.058	0.036	0.0225	0.019
1-2		0.63	0.99	0.064	0.023	0.0415	0.041
2-3		0.59	1.08	0.065	0.013	0.0521	0.056
3-4		0.59	1.09	0.049	0.025	0.0233	0.025
4-5		0.56	1.17	0.049	0.026	0.0238	0.028
5-6		0.53	1.25	0.044	0.022	0.0222	0.028
6-7		0.50	1.32	0.036	0.030	0.0061	0.008
7-8		0.49	1.36	0.030	0.032	0.0000	
8-9		0.49	1.36	0.042	0.022	0.0202	
SW-58							
0-1			0.43	0.100	0.038	0.0621	0.027
1-2			0.43	0.093	0.032	0.0613	0.026
2-3			0.48	0.099	0.045	0.0540	0.026
3-4			0.52	0.091	0.043	0.0488	0.025
4-5			0.60	0.076	0.052	0.0241	0.014

5-6	0.66	0.064	0.047	0.0164	0.011
6-7	0.71	0.044	0.029	0.0144	0.010
7-8	0.74	0.069	0.048	0.0211	0.016
8-9	0.80	0.056	0.040	0.0158	0.013
9-10	0.84	0.044	0.039	0.0057	0.005
10-11	0.80	0.039	0.038	0.0013	0.001
11-12	0.82	0.057	0.045	0.0121	0.010
12-13	0.83	0.043	0.037	0.0053	0.004
13-14	0.83	0.039	0.056	0.0000	
14-15	0.91	0.037	0.026	0.0111	
15-16	0.82	0.045	0.028	0.0171	
16-17	0.83	0.036	0.031	0.0054	
17-18	0.83	0.035	0.045	0.0000	
18-19	0.83	0.035	0.043	0.0000	
19-20	0.86	0.033	0.049	0.0000	
20-21	0.88	0.052	0.030	0.0215	
21-22	0.90	0.042	0.042	0.0005	
23-24	0.90	0.043	0.040	0.0024	
25-26	0.91	0.041	0.053	0.0000	
26-27	0.93	0.038	0.034	0.0035	
27-28	0.95	0.035	0.036	0.0000	

Supplementary references

1. Stein, R. & Macdonald, R. W. Organic Carbon Budget: Arctic Ocean vs. Global Ocean. in *The Organic Carbon Cycle in the Arctic Ocean* (eds. Stein, R. & MacDonald, R. W.) 315–322 (Springer Berlin Heidelberg, 2004). doi:10.1007/978-3-642-18912-8_8.
2. Vonk, J. *et al.* Activation of old carbon by erosion of coastal and subsea permafrost in Arctic Siberia. *Nature* **489**, 137–140 (2012).
3. Manizza, M. *et al.* Modeling transport and fate of riverine dissolved organic carbon in the Arctic Ocean. *Global Biogeochem Cycles* **23**, (2009).
4. McClelland, J. W. *et al.* Particulate organic carbon and nitrogen export from major Arctic rivers. *Global Biogeochem Cycles* **30**, 629–643 (2016).
5. Anderson, L. G., Jutterström, S., Hjalmarsson, S., Wåhlström, I. & Semiletov, I. P. Out-gassing of CO2 from Siberian Shelf seas by terrestrial organic matter decomposition. *Geophys Res Lett* **36**, L20601 (2009).
6. Alling, V. *et al.* Nonconservative behavior of dissolved organic carbon across the Laptev and East Siberian seas. *Global Biogeochem Cycles* **24**, (2010).
7. Humborg, C. *et al.* Sea-air exchange patterns along the central and outer East Siberian Arctic Shelf as inferred from continuous CO2, stable isotope, and bulk chemistry measurements. *Global Biogeochem Cycles* **31**, 1173–1191 (2017).
8. Terhaar, J., Lauerwald, R., Regnier, P., Gruber, N. & Bopp, L. Around one third of current Arctic Ocean primary production sustained by rivers and coastal erosion. *Nat Commun* **12**, 1–10 (2021).
9. Martens, J. *et al.* Remobilization of Old Permafrost Carbon to Chukchi Sea Sediments During the End of the Last Deglaciation. *Global Biogeochem Cycles* **33**, 2–14 (2019).
10. Wild, B. *et al.* Rivers across the Siberian Arctic unearth the patterns of carbon release from thawing permafrost. *Proc Natl Acad Sci U S A* 201811797 (2019) doi:10.1073/pnas.1811797116.
11. R. Lawrence, C. *et al.* An open-source database for the synthesis of soil radiocarbon data: International Soil Radiocarbon Database (ISRaD) version 1.0. *Earth Syst Sci Data* **12**, 61–76 (2020).

12. Semiletov, I. *et al.* The East Siberian Sea as a transition zone between Pacific-derived waters and Arctic shelf waters. *Geophys Res Lett* **32**, 1–5 (2005).

13. Galimov, E. M., Kodina, L. A., Stepanets, O. V. & Korobeinik, G. S. Biogeochemistry of the Russian Arctic. Kara Sea: Research results under the SIRRO project, 1995–2003. *Geochemistry International* **44**, 1053–1104 (2006).

14. Tesi, T. *et al.* Carbon geochemistry of plankton-dominated samples in the Laptev and East Siberian shelves: Contrasts in suspended particle composition. *Ocean Science* **13**, 735–748 (2017).

15. Schirrmeister, L. *et al.* Sedimentary characteristics and origin of the Late Pleistocene Ice Complex on north-east Siberian Arctic coastal lowlands and islands - A review. *Quaternary International* **241**, 3–25 (2011).

16. Treat, C. C. *et al.* Effects of permafrost aggradation on peat properties as determined from a pan-Arctic synthesis of plant macrofossils. *J Geophys Res Biogeosci* **121**, 78–94 (2016).

17. Yunker, M. B., Macdonald, R. W., Snowdon, L. R. & Fowler, B. R. Alkane and PAH biomarkers as tracers of terrigenous organic carbon in Arctic Ocean sediments. *Org Geochem* **42**, 1109–1146 (2011).

18. Lewan, M. D. Stable carbon isotopes of amorphous kerogens from Phanerozoic sedimentary rocks. *Geochim Cosmochim Acta* **50**, 1583–1591 (1986).

19. Goñi, M. A. *et al.* Distribution and sources of organic matter in surface marine sediments across the North American Arctic margin. *J Geophys Res Oceans* **118**, 4017–4035 (2013).

20. Hugelius, G. *et al.* The Northern Circumpolar Soil Carbon Database: spatially distributed datasets of soil coverage and soil carbon storage in the northern permafrost regions. *Earth Syst Sci Data* **5**, 3–13 (2013).

21. FAO/IIASA/ISRIC/ISS-CAS/JRC, 2009. Harmonized World Soil Database (version 1.1). FAO, Rome, Italy and IIASA, Laxenburg, Austria. https://www.fao.org/soils-portal/data-hub/soil-maps-and-databases/harmonized-world-soil-database-v12/en/.

22. Strauss, J. *et al.* Deep Yedoma permafrost: A synthesis of depositional characteristics and carbon vulnerability. *Earth Sci Rev* **172**, 75–86 (2017).

23. Hugelius, G. *et al.* Large stocks of peatland carbon and nitrogen are vulnerable to permafrost thaw. *Proceedings of the National Academy of Sciences* 201916387 (2020) doi:10.1073/pnas.1916387117.

24. Lantuit, H. *et al.* The Arctic Coastal Dynamics Database: A New Classification Scheme and Statistics on Arctic Permafrost Coastlines. *Estuaries and Coasts* **35**, 383–400 (2012).

25. Lenssen, N. J. L. *et al.* Improvements in the GISTEMP Uncertainty Model. *Journal of Geophysical Research: Atmospheres* **124**, 6307–6326 (2019).

26. GISTEMP Team. GISS Surface Temperature Analysis (GISTEMP). NASA Goddard Institute for Space Studies. https://data.giss.nasa.gov/gistemp/ (2016).

27. Raymond, P. A. *et al.* Global carbon dioxide emissions from inland waters. *Nature* **503**, 355–359 (2013).

28. Holmes, R. M. *et al.* Seasonal and Annual Fluxes of Nutrients and Organic Matter from Large Rivers to the Arctic Ocean and Surrounding Seas. *Estuaries and Coasts* **35**, 369–382 (2012).

29. Lobbes, J. M., Fitznar, H. P. & Kattner, G. Biogeochemical characteristics of dissolved and particulate organic matter in Russian rivers entering the Arctic Ocean. *Geochim Cosmochim Acta* **64**, 2973–2983 (2000).

30. Obu, J. *et al.* Northern Hemisphere permafrost map based on TTOP modelling for 2000–2016 at 1 km2 scale. *Earth Sci Rev* **193**, 299–316 (2019).

31. Jakobsson, M. *et al.* The International Bathymetric Chart of the Arctic Ocean Version 4.0. *Scientific Data 2020 7:1* **7**, 1–14 (2020).

32. Jakobsson, M. *et al.* IBCAO Version 4.0 Compilation Group. The International Bathymetric Chart of the Arctic Ocean (IBCAO) Version 4.0. British Oceanographic Data Centre, National Oceanography Centre, NERC, UK. https://doi.org/10.5285/a01d292f-b4a0-1ef7-e053-6c86abc0a4b2 (2020).

33. Carroll, J. L. *et al.* Accumulation of organic carbon in western Barents Sea sediments. *Deep Sea Res 2 Top Stud Oceanogr* **55**, 2361–2371 (2008).

34. Koziorowska, K., Kuliński, K. & Pempkowiak, J. Comparison of the burial rate estimation methods of organic and inorganic carbon and quantification of carbon burial in two high Arctic fjords. *Oceanologia* **60**, 405–418 (2018).

35. Lisitzin, A. P. *et al.* Comparative study of vertical suspension fluxes from the water column, rates of sedimentation, and absolute masses of the bottom sediments in the White Sea basin of the Arctic Ocean. *Doklady Earth Sciences* **465**, 1253–1256 (2015).

36. Maiti, K., Carroll, J. L. & Benitez-Nelson, C. R. Sedimentation and particle dynamics in the seasonal ice zone of the Barents Sea. *Journal of Marine Systems* **79**, 185–198 (2010).

37. Smith, J. N., Ellis, K. M., Naes, K., Dahle, S. & Matishov, D. Sedimentation and mixing rates of radionuclides in Barents Sea sediments off Novaya Zemlya. *Deep-Sea Research Part II* **42**, 1471–1493 (1995).

38. Bringué, M. & Rochon, A. Late Holocene paleoceanography and climate variability over the Mackenzie Slope (Beaufort Sea, Canadian Arctic). *Mar Geol* **291–294**, 83–96 (2012).

39. Richerol, T. *et al.* Evolution of paleo sea-surface conditions over the last 600 years in the Mackenzie Trough, Beaufort Sea (Canada). *Mar Micropaleontol* **68**, 6–20 (2008).

40. Schreiner, K. M., Bianchi, T. S., Eglinton, T. I., Allison, M. A. & Hanna, A. J. M. Sources of terrigenous inputs to surface sediments of the Colville River Delta and Simpson's Lagoon, Beaufort Sea, Alaska. *J Geophys Res Biogeosci* **118**, 808–824 (2013).

41. Kuzyk, Z. Z. A., Gobeil, C., Goñi, M. A. & Macdonald, R. W. Early diagenesis and trace element accumulation in North American Arctic margin sediments. *Geochim Cosmochim Acta* **203**, 175–200 (2017).

42. Pieńkowski, A. J. *et al.* Arctic sea-ice proxies: Comparisons between biogeochemical and micropalaeontological reconstructions in a sediment archive from Arctic Canada. *Holocene* **27**, 665–682 (2017).

43. Huh, C. A., Pisias, N. G., Kelley, J. M., Maiti, T. C. & Grantz, A. Natural radionuclides and plutonium in sediments from the western Arctic Ocean: Sedimentation rates and pathways of radionuclides. *Deep Sea Res 2 Top Stud Oceanogr* **44**, 1725–1727 (1997).

44. Baskaran, M. & Naidu, A. S. 210Pb-derived chronology and the fluxes of 210Pb and 137Cs isotopes into continental shelf sediments, East Chukchi Sea, Alaskan Arctic. *Geochim Cosmochim Acta* **59**, 4435–4448 (1995).

45. Feder, H. M. *et al.* The northeastern Chukchi Sea: benthos-environmental interactions. *Mar Ecol Prog Ser* **111**, 171–190 (1994).

46. Vologina, E. G. *et al.* Reconstruction of the conditions of Late Holocene sedimentation by integrated analysis of a core of the bottom sediments from the Chukchi Sea. *Doklady Earth Sciences* **469**, 841–845 (2016).

47. Astakhov, A. S. *et al.* Reconstruction of ice conditions in the northern Chukchi Sea during recent centuries: Geochemical proxy compared with observed data. *Quaternary International* **522**, 23–37 (2019).

48. Li, L. *et al.* Early diagenesis and accumulation of redox-sensitive elements in East Siberian Arctic Shelves. *Mar Geol* **429**, 106309 (2020).

49. Aksentov, K. I. *et al.* Assessment of mercury levels in modern sediments of the East Siberian Sea. *Mar Pollut Bull* **168**, 112426 (2021).

50. Hamilton, T. F. *et al.* Radiometric investigations of Kara Sea sediments and preliminary radiological assessment related to dumping of radioactive wastes in the Arctic Seas. *J Environ Radioact* **25**, 113–134 (1994).

51. Kenna, T. C. & Sayles, F. L. The distribution and history of nuclear weapons related contamination in sediments from the Ob River, Siberia as determined by isotopic ratios of plutonium and neptunium. *J Environ Radioact* **60**, 105–137 (2002).

52. Rusakov, V. Y., Borisov, A. P. & Solovieva, G. Y. Sedimentation Rates in Different Facies–Genetic Types of Bottom Sediments in the Kara Sea: Evidence from the 210Pb and 137Cs Radionuclides. *Geochemistry International* **57**, 1185–1200 (2019).

53. Deschamps, C.-E., St-Onge, G., Montero-Serrano, J.-C. & Polyak, L. Chronostratigraphy and spatial distribution of the Chukchi and Beaufort Sea's magnetic sediments since the last deglaciation. *Boreas* (2017) doi:10.1111/bor.12296.
54. Letaïef, S., Montero-Serrano, J. & St-Onge, G. Sedimentary processes within the Canadian Arctic Archipelago: relationships among sedimentological, geochemical and magnetic sediment properties. *Geochemistry, Geophysics, Geosystems* e2021GC009719 (2021) doi:10.1029/2021gc009719.
55. Belt, S. T. *et al.* Striking similarities in temporal changes to spring sea ice occurrence across the central Canadian Arctic Archipelago over the last 7000 years. *Quat Sci Rev* **29**, 3489–3504 (2010).
56. Durantou, L. *et al.* Quantitative reconstruction of sea-surface conditions over the last 150 yr in the Beaufort Sea based on dinoflagellate cyst assemblages: The role of large-scale atmospheric circulation patterns. *Biogeosciences* **9**, 5391–5406 (2012).
57. Ledu, D., Rochon, A., De Vernal, A. & St-Onge, G. Paly nological evidence of Holocene climate change in the eastern Arctic: A possible shift in the Arctic oscillation at the millennial time scale. *Can J Earth Sci* **45**, 1363–1375 (2008).
58. Ledu, D., Rochon, A., de Vernal, A. & St-Onge, G. Holocene paleoceanography of the northwest passage, Canadian Arctic Archipelago. *Quat Sci Rev* **29**, 3468–3488 (2010).
59. Naidu, A. S., Finney, B. P. & Baskaran, M. 210Pb and 137Cs-based accumulation rates in inner shelves and coastal lakes of subarctic and arctic Alaska: a synthesis. *GeoResearch Forum* **5**, 185–196 (1999).
60. Vare, L. L., Massé, G. & Belt, S. T. A biomarker-based reconstruction of sea ice conditions for the Barents Sea in recent centuries. *Holocene* **20**, 637–643 (2010).
