# Supplementary material for: Circum-Arctic release of terrestrial carbon varies between regions and sources
Source: Nat Commun. 2022 Oct 4;13:5858. doi: 10.1038/s41467-022-33541-0 (PMC9532443; doi:10.1038/s41467-022-33541-0)
Supplement: Supplementary file 3 — Description of Additional Supplementary Files [file 41467_2022_33541_MOESM3_ESM.pdf]

Supplementary Data 1: End member database
